# Operationalizing stakeholder engagement for gene drive research in malaria elimination in Africa—translating guidance into practice

**DOI:** 10.1186/s12936-022-04241-3

**Published:** 2022-07-23

**Authors:** Lea Pare Toe, Bakara Dicko, Richard Linga, Nourou Barry, Mouhamed Drabo, Naima Sykes, Delphine Thizy

**Affiliations:** 1 Institut de Recherche en Sciences de la Santé, Ouagadougou, Burkina Faso; 2 Malaria Research and Training Center, Bamako, Mali; 3 Uganda Virus Research Institute, Entebbe, Uganda; 4 Imperial College London, London, UK

**Keywords:** Stakeholder engagement, Community engagement, Gene drive, Vector control, Malaria, Responsible research, Co-development, Genetic approaches

## Abstract

Gene drive mosquitoes are increasingly considered a potential transformational tool for vector control of malaria mosquitoes. As part of efforts to promote responsible research in this field, a number of guidance documents have been published by the World Health Organization, National Academies and expert groups. While virtually all recent guidance documents on gene drive research stress the importance of stakeholder engagement activities, no specific guidelines on implementing them have been established. Target Malaria, a not-for-profit research consortium developing a vector-control gene drive approach to eliminate malaria, has reflected on how its stakeholder engagement strategy translates engagement guidance documents into practice. The project analysed and addressed the tension between the context specificities and the international recommendations. The engagement strategy combines published recommendations for responsible gene drive research, information collected from the local context where the project operates and a set of principles guiding the choices made. This strategy was first developed during the early phases of the project’s research, years ahead of any activities with gene drive mosquitoes in those countries of operations. These earlier activities, and their related engagement, allow the project to develop and adapt an engagement strategy appropriate for potential gene drive research in its field site countries. This paper offers a description of a stakeholder engagement strategy operationalization based on (1) adaptation to stakeholder preferences, (2) inclusiveness and (3) empowerment and accountability. The authors hope to offer concrete examples to support other projects with the development and implementation of their engagement strategies with particular attention to the co-development principle.

**Supplementary Information:**

The online version contains supplementary material available at 10.1186/s12936-022-04241-3.

## Background

The ongoing Covid-19 pandemic underscores the importance of the participation of communities and patients in public health interventions [1]. Studies of the implementation of Covid measures [2, 3], have confirmed insights gained from other recent epidemics such as HIV/AIDS [4] and Ebola [5].

Based on these insights, policymakers have worked to integrate community participation into global health guidelines [6]. The World Health Organization (WHO) guidance on the ethics of vector-borne diseases dedicates a full section to community engagement [7]. In the same document, the WHO draws attention to the importance of an "ongoing process of community engagement […] to ensure that studies respond to questions of local public health importance", highlighting the particular importance of doing so for "new technologies". The document goes on to single out genetically-modified vector control research and gene drive in particular. New questions are raised: are potential applications for gene drive vector-borne diseases or invasive alien species management [8] significantly different from other interventions from an ethical perspective? Do they create new engagement imperatives? Or does this new research area merely offer an opportunity to rekindle discussions about future beneficiaries’ involvement in public health research?

This paper is a response to a call from the WHO to document "existing community engagement strategies and their impact […] in order to share relevant best practices within and between countries" [34]. This call echoes other calls from academics and from other stakeholders interested in gene drive research to share the existing experience from engagement practitioners.

Target Malaria engagement team decided to articulate the specific approach that they took to elaborate and adapt the stakeholder engagement strategy since the initiation of this work. Because of the project's distinctive nature and the communities it partners with, this stakeholder engagement strategy is not intended to serve as an example for other projects to replicate. However, it may offer a case study from which other vector control research projects can consider their approaches to community partnerships. The supplementary material provides a detailed review of how recommendations can be implemented during a research project, articulating the general project approach and providing whenever possible a concrete example from one of the Target Malaria’s countries of operation. In addition, this paper proposes some more in-depth descriptions of recommendation operationalization, potential challenges therein, gaps in the emerging guidance documents that would benefit from further social sciences research, and an exchange among practictioners involved in similar projects of community practices. This responds to comments about the challenges of operationalizing the existing guidance and bridging general principles with concrete implementation methods [35].

By doing so, the authors hope to contribute more broadly to the development of public health research approaches that place the potential beneficiaries at the centre of the process. The scope of this approach is focused on research stages which would include the potential releases of gene drive mosquitoes, but not its full roll-out as a public health intervention.

### Gene drive for malaria control

Gene drive is a naturally occurring phenomenon that favours the inheritance of certain genetic traits, "resulting in the gene becoming more prevalent in the population over successive generations" [9]. The mechanism can be harnessed to increase the frequency of a particular gene or genes in a wild species' targeted population. For instance, one can 'drive' or cause a sustained increase in frequency of a gene that eventually leads to the reduction of a disease-vector population. By developing gene drive *Anopheles gambiae* mosquitoes, Target Malaria researchers aim to complement vector control tools already implemented by national malaria control programmes [10]. The approach constitutes a new class of vector control interventions: an intervention using genetic modification aimed at a public health outcome in the environment. As such, it falls under several international oversight frameworks that either take into consideration its genetic modification specificity from a biosafety perspective (as is the case with the Cartagena Protocol) [11] or its potential health impact (as is the case with WHO oversight) [12]. National regulations operationalize the global oversight mechanisms for instance with specific risk assessment frameworks. For researchers, this overlap between different frameworks can be complex to navigate[13].

The involvement of potential future beneficiaries in the research is part of these existing guidance materials. While ethical arguments surrounding such involvement are not new[14], new technologies and new disease outbreaks [2] have led to a renewed focus on this topic. This focus provided an opportunity to review practices and to establish guidelines on responsible and effective stakeholder engagement. The resulting studies [15–19] and guidelines [7, 8, 20–22] promote different participation levels as set out by the established spectrum for participation [23]: information, consultation, involvement, collaboration, and empowerment.

The literature on engagement for gene drive promotes active participation. However, few practical recommendations explain how to achieve such participation, when to engage, which modalities to use. In the absence of precedents for engineered gene drive organisms’ release, there are no existing practical examples on how to responsibly and effectively engage about this topic. In these circumstances, stakeholder engagement practitioners must establish how those general theoretical recommendations can be translated into practice and have to establish the legitimacy for their proposed models of engagement. There are comparative precedents—from the area-wide vector control field [17] (such as the release of *Wolbachia* infected mosquitoes [16, 24, 25], sterile male insects, or genetically modified sterile mosquitoes) or biocontrol field [26, 27]—that can be looked at to identify good practices and evaluate if they are appropriate for this new field.

Target Malaria is one of the leading projects working on gene drive for malaria vector control. Its mission is to "develop and share new, cost-effective and sustainable genetic technologies to modify mosquitoes and reduce malaria transmission" [28]. Target Malaria operates in four countries: Burkina Faso, Ghana, Mali and Uganda. Engagement is a central pillar of the project, together with science and regulatory affairs [29]. Target Malaria is progressing through several phases of iterative research to enable its stakeholders and national authorities to approach this new field of research and its potential in a progressive manner. This approach draws on the guidance developed by the WHO [20] as well as views from experts [8, 21]. This research pathway is re-evaluated continuously based on the information gathered from the research and advances made by other research teams and new guidance from authorities and experts, including the feedback from stakeholders. Similarly, the early phases of stakeholder engagement are critical to develop the future engagement and community agreement model that will be used for potential gene drive evaluation.

**Fig. 1:**
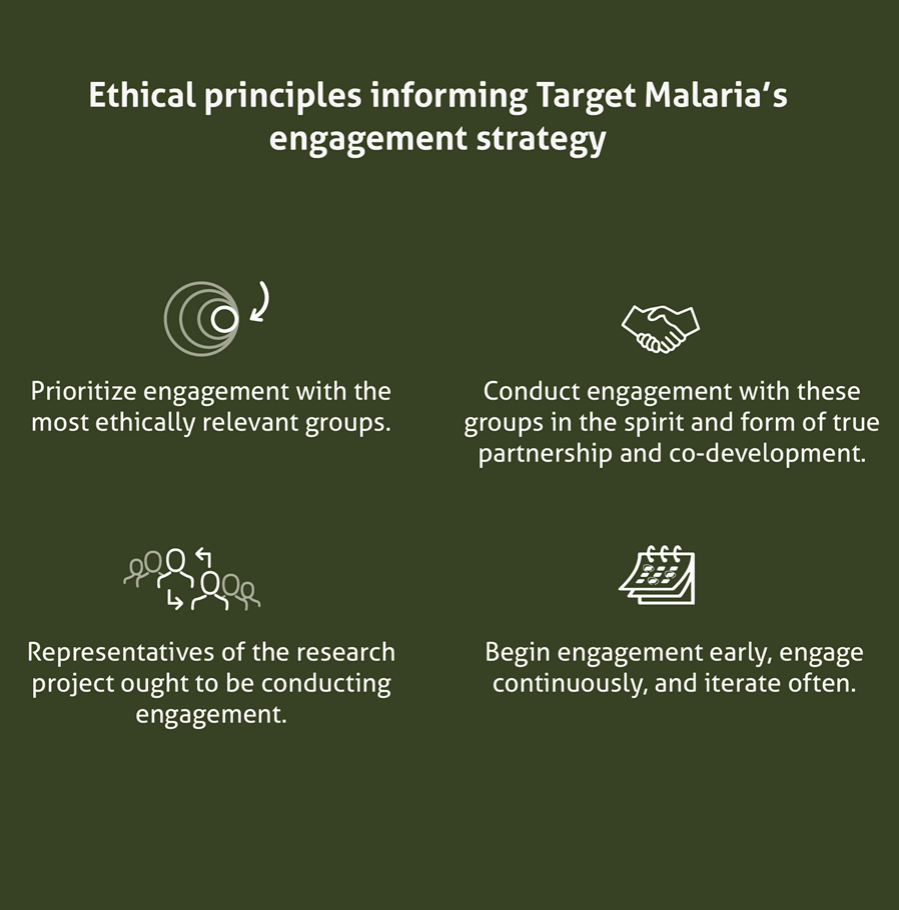
Ethical principles informing Target Malaria's engagement strategy [32]

Early on, the project examined two clear perspectives on engagement: the 'utilitarian perspective', where "Interventions that are based on a utilitarian perspective seek to involve communities in order to improve the effectiveness of the intervention", and the 'social justice perspective', where "community members are empowered to determine for themselves the priorities and ways in which they want service resources to be deployed" [30]. The project has aimed to reconcile the two perspectives, as proposed by Popay [31], recognizing the need to empower potential beneficiaries and stakeholders to co-develop the approach, while recognizing that the engagement is also a way to improve the intervention and to build a dialogue to increase acceptance of the technology. This positioning is deeply rooted in the ethical principles (Fig. 1) of the project's engagement strategy [32].

In 2014, when the project started working more intensively on its engagement approach, the WHO guidance framework for testing genetically modified mosquitoes [20] was the principal guiding document. In this document, the WHO defines engagement as "practices undertaken to inform stakeholders about the diseases and vectors of interest and goals of a proposed research study or intervention trial, and to understand their perspectives and reaction". While this document provided useful support, particularly regarding the inadequacy of an individual consent process, it focused on engagement's utilitarian perspective. Therefore, it lacked specific guidance for the project to empower potential beneficiaries and stakeholders to participate actively in the research process. The project identified early on that co-development—defined as "a collaborative process of jointly designing a research pathway and its resultant intervention to reach a common goal" [33] was at the heart of its approach.

**Fig. 2:**
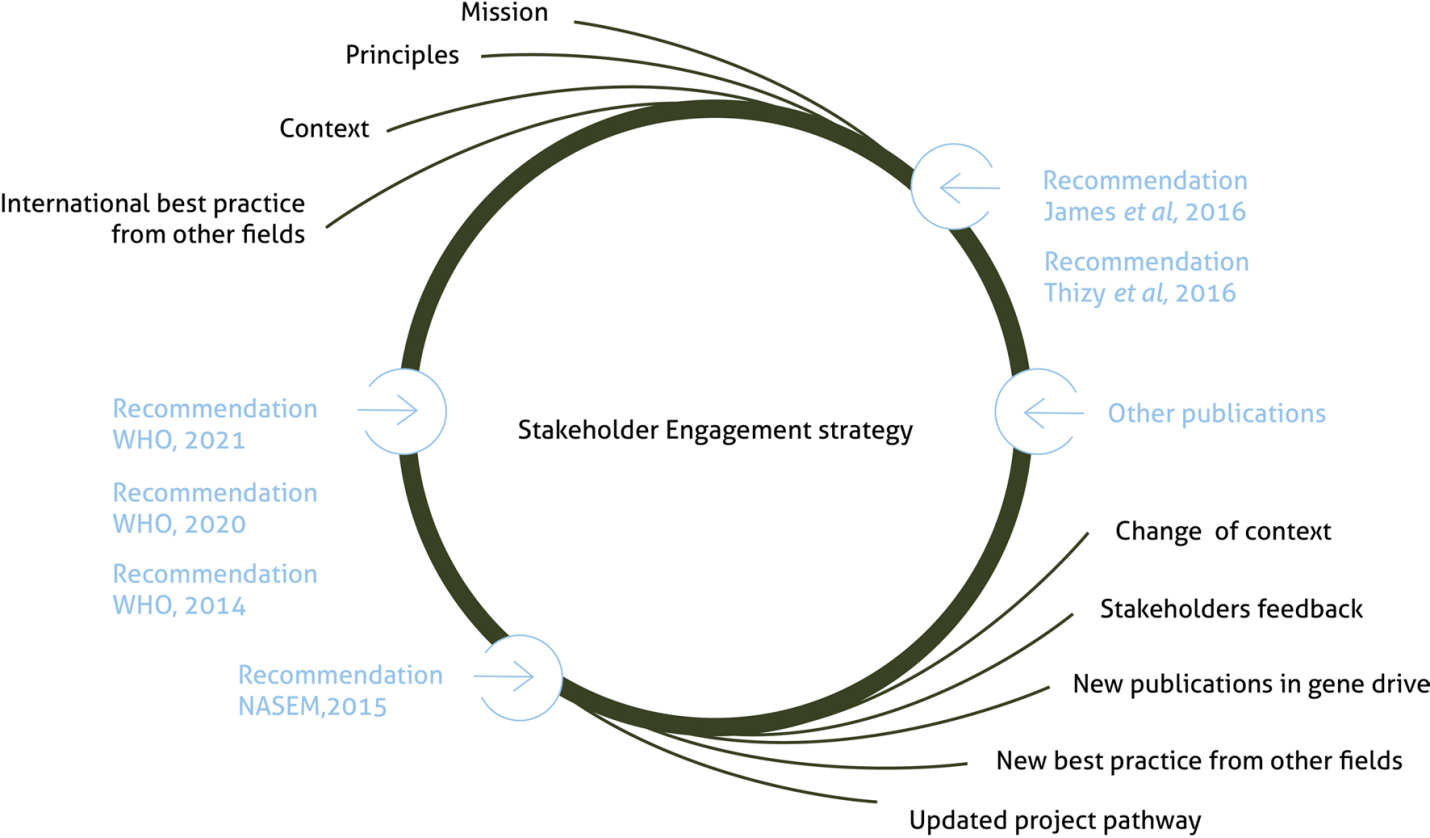
The evolution of Target Malaria’s engagement strategy: an iterative process

The Target Malaria engagement team developed its strategy considering the context, its ethical principles for engagement, and the 2014 WHO guidance framework. The strategy was conceived as an evolving document, to be nourished by a changing context, stakeholder feedback, potential changes in the project's implementation, and new guidance documents and relevant publications (Fig. 2).

The above-described process requires a high degree of self-reflection on one’s practice and experience and flexibility towards changing the strategy if needed. Besides, with the emergence in recent years of new guidance documents [7, 8, 21, 22, 33] and literature on other area-wide interventions [16, 25], the balancing exercise between local context specificities and international norms or recommendations revealed a crucial part of engagement strategy design and implementation. As Fig. 2 demonstrates, the strategy is also reviewed when the project pathway evolves, and for instance when the project moves from one phase (such as non gene drive genetically modified sterile male mosquitoes) to another phase (such as non gene drive genetically modified male bias mosquitoes) [34].

### Contexts of operations

Target Malaria operates in countries whose populations are severely affected by malaria. Out of its four partner countries, Target Malaria currently plans to evaluate its genetic approach to vector control in three: Burkina Faso Mali and Uganda; Ghana is presently focusing on complementary studies on mosquito ecology. The three countries chosen for the technology evaluation accounted for 11% of the global malaria infection cases in 2019 and are part of ten countries with the highest malaria rates worldwide (5% in Uganda and 3% each in Burkina Faso and Mali) [36]. Due to the high number of infections and its burden on public health facilities in these countries, malaria is considered one of the main threats to public health [36], even in the context of the COVID-19 pandemic [37].

In line with its mission, Target Malaria operates as a network of partners that include some of the most active public health research centres in these malaria-endemic countries. In Burkina Faso, the research is implemented in collaboration with the Institut de Recherche en Sciences de la Santé (IRSS), in Mali with the Malaria Research and Training Center (MRTC), within the University of Sciences, Techniques and Technologies of Bamako, and in Uganda with the Ugandan Virus Research Institute (UVRI). In all three cases, the institutions host Target Malaria's insectary work [38] located in urban areas of Bobo-Dioulasso for Burkina Faso, Bamako, Mali, and Entebbe in Uganda. The neighbourhoods where the facilities are in Bamako and Bobo-Dioulasso present similar legacies of traditional village structures, though those have been adapted to their urban settings. In Uganda, the research facility is in a very urban environment characterized by the absence of more traditional forms of governance and realities of very active and fast-paced urban life. There are currently no genetically modified mosquitoes in Uganda.

In addition to the research facilities within the partner institutions, the project also operates in villages, where field-entomological activities are taking place—to understand the mosquito population better and to evaluate the different phases of the technology being developed by Target Malaria. This step is critical for the research on gene drive as it allows, amongst other things, to create models that can simulate the potential dissemination of gene drive mosquitoes [39]. Although the field research areas are located relatively close to the insectary facilities (less than 50 km), their populations and compositions vary. For instance, in Burkina Faso, villages are characterized by the presence of two main groups: those who founded the village and as such are recognized to have customary rights on the land, and those who settled in the village at a later stage, which can be from different ethnic and linguistic groups [40]. Although those who settled in the village later on participate actively in the decision-making process concerning village life, the official power structures are dominated by the founders' group. In Mali, the village population is more homogeneous with a shared history and language. In Uganda, the project has worked with a wider variety of villages over time, investigating mosquito populations on mainland villages and the Lake Victoria islands. The current working sites on the islands are characterized by a sizable population of migrant fishermen who are temporary dwellers of the villages and can come from various locations.

Civil society is also an essential part of the landscape where the project operates. The three countries boast a vibrant civil society sector, contributing to various sectors, such as economic development, health, social, cultural or religious affairs. Civil society groups are often organized in networks, or coordinate along themes (e.g., around economic development) or by composition (e.g., women's association coordination). While they are more visible at the national level, there are also regional civil society networks in secondary cities or towns and a network of relationship with more informal groups at the local level.

The project started the partnership with African research institutions in Burkina Faso, Mali and Uganda in 2012. From the onset, community engagement was part of the activities in-country, mainly focusing on information and consent process for routine mosquito collections in the villages. In 2014, In Burkina and Mali, as the project started more actively preparing for the first phase of work with non gene drive sterile male mosquitoes, the engagement team began elaborating its strategy, years before any genetically modified mosquitoes were imported or released in partner countries; while in Uganda the work initially focused on mosquito population characterization and the engagement surrounding the insectary construction. This early engagement contributed to the entomological studies for the characterization of mosquito populations [41, 42], the importation and contained use [43] of this sterile male mosquito strain [44] in Burkina Faso in November 2016 and Mali in September 2019, the small-scale release of 6,400 genetically modified non gene drive sterile male mosquitoes in one of the villages in Burkina Faso in July 2019, and the inauguration of the contained use facility in Uganda in July 2019. In addition to communities' agreement, all importations, contained use and small-scale release of genetically-modified mosquitoes had received prior regulatory approvals from the authorities of the country where they took place.

## Methods

This paper is the result of an unusual method. It does not result from a research study as such, nor does it describe the results of a specific protocol. Rather, it intends to provide a review of how Target Malaria elaborated and adapted its engagement approach, guided by its ethical principles [32] guiding [32] its response to[32] new guidelines and recommendations and the specific local context in which the project operates.

As highlighted in the WHO guidance for vector control "*the guidance cannot offer universally applicable answers to the complex ethical issues raised, nor can it provide a checklist of issues that are necessarily relevant in all situations. Rather, its goal is to help readers recognise aspects of their work that raise significant ethical challenges and to respond to these challenges in accordance with internationally accepted values and norms*" [7].

This paper was prepared in three steps.

### Step 1. A systematic response to the recommendations from existing guidelines

The authors focused on six key publications: three from the WHO [7, 20, 22], one from the National Academies of Science, Engineering and Medicine [8], and two from expert groups[21, 33]. These papers were selected on the following criteria: (1) specific to gene drive research for vector control or area-wide vector control research, (2) covering stakeholder engagement aspects broadly and not only the question of consent or community agreement, (3) providing recommendations or guidance to a search in academic publication databases using the keywords "gene drive", "vector control", "genetically modified mosquitoes", and "engagement". Additional file 1: Table S1 presented in supplementary material analyses the different sources that authors considered and shows how authors applied the criteria to inform the selection. Once selected, one of the authors listed all recommendations and tried to group them by themes. Then, the authors discussed how the project’s engagement strategy was guided by the early days recommendations and how the overall engagement strategy designed by the project responds to recommendations that might have been published after the strategy was designed, and how the strategy was informed by some of those emerging strategies (Fig. 2). Through these discussions, they identified some tension points, where their practice was confronted to guidelines or recommendations that were either vague and therefore with limited value, or difficult to implement as such in their operating context. Finally, the authors shared the themes between themselves and provided concrete example recommendations implementation in their context. The result of this process is presented in Additional file 1: Table S2.

### Step 2. Identify the key considerations that guided the translation of the recommendations into practice

The authors had a series of discussions to confront their experience between the different countries and the examples described during Step 1. These discussions were particularly focused on the areas of tension, where the recommendations implementation was not straight forward. From these discussions, the authors identified a series of key considerations that had guided their work in how the recommendations were translated into practice and how they would decide between competing perspectives when the recommendations conflicted with the context.

### Step 3. Identifying illustrating examples

The authors decided to identify specific examples from Burkina Faso, Mali and Uganda illustrating where the strategy confronts the recommendations, principles, context, and stakeholder feedback and demonstrating how the strategy remains flexible to adapt to the context and stakeholder feedback while responding to the ethical challenges raised in the recommendation documents. To illustrate the balance between these different elements, case studies are described with more details (Textboxes 1, 2, 3 and 4). The case studies were chosen to show practical engagement modalities responding to ethical requirements and guidelines and reflecting the diversity of engagement activities.

### Definitions

**Fig. 3:**
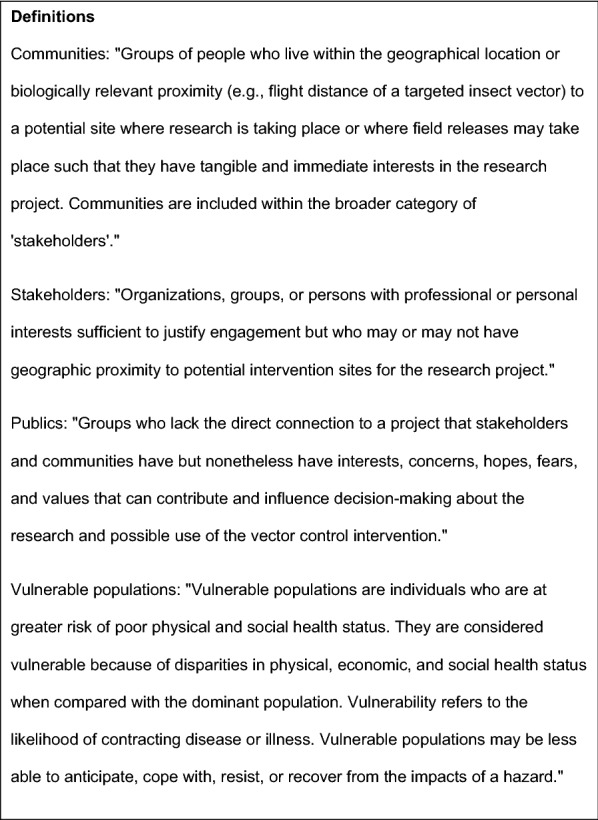
Definitions

This paper uses the definitions of communities, stakeholders and publics adapted from the definitions proposed by the National Academies of Science Engineering and Medicine [8]. They are presented in Fig. 3 for readers' reference, together with a definition of vulnerable populations [45].

## Results

### Key considerations for operationalizing stakeholder engagement

Three key considerations were taken into account for operationalizing stakeholder engagement when translating the guidance into practice: (a) adaptation to stakeholders’ preferences, (b) inclusiveness and (c) empowerment and accountability.

### Adaptation to stakeholders’ preferences

The project has built its stakeholder engagement approach by adapting to the context and values of stakeholders. This approach is deeply rooted in the principles of co-development as well as engaging early and continuously[32]. In the absence of set rules, or established best practices, the engagement strategy was built on stakeholders' preferences and continuously adapted to their feedback and the changing context. This approach recognizes their role in establishing a dialogue on the research that could be satisfactory to them.

The importance of this context-specific approach was later highlighted in the subsequent guidance documents that were published. For instance, the National Academies of Sciences Engineering and Medicine in its recommendation 7–6 indicates the need to "*adopt engagement plans that are relevant to the social, cultural, and political contexts in which gene drive research may be planned*" [8], while in its second edition of the Guidance on testing genetically modified mosquitoes, the WHO makes an explicit reference to the value of a “*co-development approach that emphasizes authentic partnership and knowledge engagement*” [22].

Nonetheless, several recommendations from key guidance documents raise tensions as they are not necessarily culturally appropriate or adapted to the context. For example, some recommendations refer to “household consent” for activities involving direct participation to data collection (such as field entomology). In their response, the authors highlight the fact that the concept of household is not necessarily appropriate in their context of operations in West Africa and that they had to balance the recommendations and the context and stakeholders’ preferences (Additional file 1: Table S2).

This adaptation can challenge existing practice and require other project functions to reconsider their approach, as illustrated in the Textbox 1 about the ‘scientific café experience’ in Mali. In that activity, the project integrated the community's request to replicate an activity with another group of stakeholders, which required significant adaptations—for instance, translation to the local language, and training of scientists to avoid jargon. Looking forward to engagement for gene drive field evaluations, this flexibility will be even more critical considering that a broader set of stakeholders and communities will need to be engaged. Within the same project, and the same country of operations, models will likely vary to take into consideration the diversity of perspectives and preferences from stakeholders.

### Textbox 1: Responding to requests of stakeholders. A case study from the scientific cafe in Mali

Mali has a tradition called the 'café citoyen' ('citizen café'), where citizens can talk with their leaders and elected officials, taking place in urban settings and often broadcast on national television. In the same spirit, the national public health institute (INRSP) has been organizing scientific cafes to foster exchanges between scientists. The Target Malaria team in Mali thought that this model could offer an excellent opportunity to increase the dialogue within the University of Sciences, Techniques and Technologies of Bamako and the MRTC where the research facility is located, to increase the knowledge and dialogue among other researchers. The first event was a success because of the high attendance and the calls from participants to replicate this opportunity for an exchange to discuss new aspects of the research. When the project mentioned this event to the community living around the research facility, community members requested similar events, open to a broader public of non-scientists, be organized. The project responded to this request by organizing another session open to the public and inviting leaders and local community members.

The format of these events involves a small number of people (up to 30 participants). For the café with scientists, an invitation had been sent to all the university research units that then nominated participants according to their interest and availability, while for the public café invitations were sent to different community leaders who discussed and decided who should participate. All participants are on the same level (sitting in a circle rather than the more usual theatre set-up of academic presentations), which helped foster an open dialogue between the different participants. The second event, open to the public, was an opportunity for other scientists from MRTC who are not part of Target Malaria to appreciate the impact of having direct contact with communities and some of the challenges of such interaction. For instance, in this second event, the use of the local language (bamanakan) and the need to avoid scientific jargon to establish a common understanding were a new experience for many researchers.

The participants thanked the project for organizing an opportunity to meet scientists directly, interact with them regarding their expectations and concerns, and get a direct appreciation of ongoing research. This feedback was expressed during the event itself when participants took the floor but also in subsequent engagements with the community when they shared their experience with other members who had not participated. It was also an opportunity for stakeholders to discuss gene drive with a broader group than the stakeholder engagement team from Target Malaria. This type of format helps to demystify the science and breaks down some of the barriers between community members and scientists who are often perceived as part of a disconnected elite. The stakeholders expressed their desire for additional scientific cafes to discuss other themes, such as biosafety. The project plans to organize these as soon as it is feasible.

In some cases, the feedback from stakeholders directly impacts other functions of the project, such as the process of obtaining consent for routine field entomology collections. These collections check mosquito population density, their genetic composition, provide sources of local mosquitoes for insectary colonies, and investigate associated insects in the vector habitats as background on the area's ecological conditions. Initially, the project had requested consent for each mosquito collection session using the "insecticide spray catch" technique before each operation, taking place at that time every month. This meant that the team would re-explain the procedure every time and read the information sheet to the participant to ensure that the person consenting had understood the procedure and any potential risks and benefits, following the usual ethics protocol and practices at the research centres. However, because the project was doing monthly collections for several years, participants expressed fatigue with the process and discomfort with the number of documents they had to keep (each consent meant a copy of the information sheet and the consent signature form). After consultation with various stakeholders (village representatives, institutional ethics committees and the ethics advisory committee of the project), the decision was taken to implement an annual written consent, with a verbal consent for each collection, to remind the participants that they have the right to withdraw from the process at any point. The ethics protocol for each institution was adapted accordingly. This was also an opportunity for the project to reflect on ways to improve the information process accompanying the consent, to make it more engaging while ensuring that the consent provided was sufficiently informed.

Anthropological studies provide useful indications on community dynamics, social systems, and motivation to participate in the research [40]. Still, direct observation and real dialogue with stakeholders allow for the strategy to be co-developed through continuous iterations and remain dynamic to integrate changes in preferences or lessons learned. It also allows the project to have local specificities, co-developing activities closer to communities and finetuning strategies to sub-national and even sub-regional particularities. This is particularly important in a project like Target Malaria, set up for an extended period. The long duration of the research can occasion fatigue. This fatigue can manifest itself in reluctance to participate in research or engagement activities. This fatigue can be occasioned by the recurring engagement and research activities requiring communities’ participation, which can be time consuming over the years. The authors have experienced how community members can express fatigue when being engaged or consulted about the same project, albeit for different activities or phases of work. This fatigue is also increased by the fact that while this research is taking place, there are no immediate impacts on the malaria prevalence in these communities. The communities’ investment in time and attention is based on the hope that this research can be successful in the future as highlighted by Barry et al. [40].

Remaining open and flexible to adapt to stakeholder preference [46] has been a successful way for the project to maintain a good relationship with the communities where, in some cases, it has been operating for eight years. However, the balance between proactive engagement and over-engagement creating fatigue is a complex one. The project continues its reflection on how to avoid potential negative impacts of the engagement activities. This remains an under-research area, and to this day, guidance documents do not explicitly address these.

By integrating stakeholder feedback and proposals, the project ensures that the contextualization of its engagement strategy is adequate—as recommended by most guidance documents. However, the adaptation to stakeholders’ preferences—which are informed by their socio-cultural norms—can raise some tensions when implementing guidance recommendations as highlighted in several instances in Additional file 1: Table S2. Such tensions often raise discussions, in the literature, amongst experts, but also more generally in the public, about what should take priority between the guidance implementation and the cultural preferences and context specificities.

### Inclusiveness

Target Malaria's mission to "develop and share new, cost-effective and sustainable genetic technologies to modify mosquitoes and reduce malaria transmission" [28] poses a critical challenge for engagement: ensuring that populations affected by malaria, who are in the majority rural and poor populations [47], be equipped and empowered to decide on an innovative genetic approach. While this is not unique to gene drive research, concerns about power dynamics involved in this research field have been expressed and highlight the need for this inclusiveness [35]. As a consequence of the project's focus on directly affected populations, and a recognition of potential power dynamics in which those population might not be empowered for decision-making, inclusiveness has been a driving factor in the stakeholder engagement. As such, the project promotes the participation of all categories of stakeholders including the vulnerable groups (such as women, youth, elderly, people with disabilities) that could be affected by the project's work. While a uniquely utilitarian engagement strategy dedicated to "securing acceptance" for the technology could have focused only on stakeholders capable of influencing the policymakers or the decisions of the affected communities, Target Malaria decided to add an inclusive approach incorporating the social justice imperative and engaging directly affected communities and communities surrounding its facilities. This approach echoes the WHO call for intersectional analysis to "more targeted interventions and policies in complex real-world settings" [7]. The recommendation can similarly be applied to engagement activities, to avoid information and consultation activities perpetuating existing inequality dynamics.

For the example of the inauguration of the Ugandan insectary (see Textbox 2), having an inclusive strategy meant tailoring the event's format to the stakeholders' needs, which meant a more significant engagement effort for the whole team, including laboratory staff in charge of insectary visits.

### Textbox 2: Pursuing inclusiveness by adapting the engagement process. A casestudy from the insectary inauguration in Uganda

In 2019, the Target Malaria insectary construction was finalized in Uganda. The project was keen on organizing an inauguration to open its new facility to local stakeholders and celebrate a significant milestone.

Before the construction, engagement efforts had allowed the project to take stakeholders' perspectives into account, for instance, by addressing neighbours' discomfort with the noise generated by the construction site, by restricting it to weekends as suggested in the feedback.

Building on this pre-existing dialogue, the Uganda Target Malaria team wanted to ensure that the insectary inauguration could be inclusive and responsive to the requests for continuous openness as expressed by stakeholders who were interested in seeing the activities taking place in the research centre. Thus, the inauguration of the insectary was phased into three activities to enable various stakeholders' effective participation. The first inauguration activity was attended by national-level stakeholders who comprised a representative of H.E. the President of Uganda and representatives of different government authority levels, the district where the project operates, and representatives of other key partners working in the fields of health and research. This activity involved formal scientific presentations about the project and dialogue with the stakeholders. A second inauguration activity was organized for the local community stakeholders around the insectary to enable people to interact directly with the project staff and visit the new facility before it began its containment operations. A third activity was dedicated to wider civil society stakeholders to share knowledge, promote dialogue, and gather feedback. Finally, through the engagement of key national media outlets, the project reached a broader audience, sharing essential information about its facility and research.

The diversity of formats and engagement activities for this inauguration corresponded to the wide range of interests and expertise within these groups. For instance, while authorities were interested in the big picture questions about the role of Uganda and UVRI in vector control 10.1186/s12936-022-04241-3 innovation and the inauguration of a new kind of facility in the country, the communities living around the insectary had more specific questions about the containment measures and future activities in the lab. The engagement activities also helped ensure that community and civil society members found an environment conducive to dialogue with the research team, as opposed to their voices being lost in a large event with officials where the scientists would not have been able to devote their full attention to them. This was done by creating small groups that could have a more direct and informal discussion with the researchers. The participants were very engaged during this event, asking numerous questions to the scientists and project team.

When it comes to how communities should come to and express their decision about area-wide vector control research, the question of who is making in this decision, how inclusive that decision is, and whether those making the decisions are sufficiently empowered is central [48]. In response to these questions, the project co-developed a community agreement model with the community members. When it was initiated, this responded to the WHO recommendation of "Determining what the community wants/expects in terms of engagement or consent" [20]. More recently, the WHO guidance on ethics for vector control confirmed the importance of working closely with communities, stating, "Because the nature of community authorization will vary in different contexts, it is essential to investigate what the community itself considers to constitute a valid authorization" [7]. In 2021, the WHO highlighted that the process “will depend on the values, goals and preferences of the community” [22].

When consulting communities about appropriate community agreement models, Target Malaria was confronted with a double ethical imperative: respecting existing governance systems and cultural norms on one side and aiming for inclusiveness on the other [49]. This is a representative example of the potential tension between the local context and a more theoretical guidance framework. For instance, the WHO guidance on ethics and vector-borne diseases identifies several social determinants of vector-borne disease (gender, age, socioeconomic status, migration status, indigenous people and communities) and calls upon researchers to address those determinants as a “matter of social justice” and include “safeguards and protections [...] to avoid exacerbating them” [7]. In many traditional societies where the project operates, the decision-making power is held by the dominant ethnic group, usually represented by a male chief, who is usually also an elder of the community [50, 51].

There is, therefore, a tension between a rigid interpretation of this principle of inclusiveness and the local cultural norms. Aligned with its value of co-development, the project consulted the community to understand how decision-making processes should reflect the community decision while understanding at the same time what safeguards could be put in place to protect these vulnerable populations. In addition to this, the project consulted those not represented in the decision-making systems—for instance, because they do not belong to the founders' group having customary land rights and therefore not being represented in the village chiefdom, and women who are also often excluded from these decision-making groups (especially in the West African countries where the project operated)—to get a validation of the process and ensure its legitimacy (see Textbox 3 and Pare Toe et al. [52]).

The teams also facilitated mechanisms to ensure that those other voices could express their concerns, expectations or feedback to the project. For instance, when creating accountability mechanisms (see Textbox 4), the Burkina Faso team encouraged the participation of more vulnerable groups in the committee in charge of monitoring the project's activities. It led to the integration of women and youth representatives in the monitoring committee. This process also identified specific engagement activities organized to foster the participation of vulnerable groups (such as women for instance) who might not attend the main meetings—because of limited availability due to their housework—or who might not express their opinions in front of men. When asked about what might bring them to engagement activities, women mentioned that having more interactive formats would help them to prioritize this activity, which led the team to organize small quiz games for example. In this whole process, the team maintained a close engagement with the community leaders to explain the rationale for broadening participation and ensuring that this is acceptable to them and not perceived as questioning their traditional norms and culture. In these cases, the leaders were very supportive of this process as they thought it was important to ensure that all community components were informed and engaged about this project.

### Textbox 3—Strategy for ensuring representation in decision-making

In the rural area where the project operates, the traditional local system of organization favours the delegation of power, particularly for social affairs, to a small number of leaders. The community delegates its decision-making capacity to them for issues relevant to the community as a whole. Target Malaria's activities—except for those taking place directly in individuals' homes—fall under this category.

Even if the engagement is inclusive and the project multiplies opportunities for a broad range of voices to express themselves—through focus groups, individual meetings, grievance management mechanisms—these leaders continue to play an essential role in the engagement process, notably because they often express the community decision about processes, preferences, or are consulted first about the proposed approaches and activities. Hence, it's crucial to ensure that the process is aligned with the principles of social justice and that it does not silence vulnerable groups or minorities; in short, that it truly represents the community in all its diversity and acts on behalf of the community.

The first step to verify this is to obtain an in-depth understanding of the community's social organization and governance system. To this end, the team spearheaded an ethnographic study based on a qualitative approach to understand the social fabric and dynamics, including the relationships between the different components of the community (be they based on gender, ethnicity, age, interest) and to understand decision-making processes for community affairs. Investigating whether community representatives genuinely represent the community is central to this task. For example, in Burkina Faso, this anthropological study was designed, implemented and analysed by the project engagement team that includes both engagement practitioners and social scientists [52]. Carrying out the ethnographic study with a broad range of individuals allows one to cross-check information and to identify inconsistencies in the representative system or disputes regarding leadership legitimacy.

Once the community's governance landscape is established, the project validates the findings with a selected sample of community members. This step ensures that the interpretation of the findings is appropriate and reflects the community structure and preferences. Verifying this legitimacy does not stop there; it is a continuous process and integral to the engagement work. The team meticulously analyses community feedback, complaints, or possible disputes about appointed leaders' 'representativeness'.

These efforts to promote inclusiveness require consultation with vulnerable groups to understand what instruments can facilitate active and open participation. In some cases, it might only need a different timing respecting their workload; in other cases, it requires a different meeting format (e.g., door-to-door meetings instead of larger gathering). In Uganda, it was found that the organization of activities such as the "Test and Treat Day" were useful in reaching out to groups who might be more distant from the public health discussion. The Test and Treat Day is an activity where the project works with the local health centres to provide a malaria testing opportunity and treatment (treatment decision is made according to National Malaria Control policy). Information on malaria transmission and prevention methods and the Target Malaria research are provided at the same venue. The commitment to inclusiveness requires "going the extra mile" to address potential marginalization, and an acute awareness from team members to identify potential marginalization factors.

Part of the project's commitment to inclusiveness is fostering dialogue with critical voices that might have concerns or even oppose the early research phases or the future deployment of gene drive technology for malaria control [49]. While they might not be directly affected by current research activities, engaging these stakeholders is an ethical imperative in applying the project's values of openness and accountability and being encouraged in recommendations that either pre-existed this strategy [20] or were subsequently developed [22, 33]. The engagements are not aimed at convincing critical voices, but to share evidence-based information, while hearing the concerns of stakeholders (which may inform the design of additional activities to obtain relevant information), and in some cases acknowledging differences in values. As such, the teams have opened their facilities to visits from critical groups, for instance in Burkina Faso before the contained use of non gene drive sterile male mosquitoes began in the insectary and before the nationally approved small-scale release into the environment, or in Uganda during the inauguration of the insectary.

In Burkina Faso, a mechanism was put in place with stakeholders to facilitate this dialogue: the "relay group" for civil society. This group was created in 2017 to frame structured interactions with civil society representatives, including groups who are critical about this research. The group established its working process by developing a "charter" signed by all participants and establishing the roles and responsibilities of participants and the project. In 2019, two member organizations left the group following the approved release of sterile male mosquitoes, including the person appointed as coordinator for the group by his peer members. Since its initiation, the two organizations had been active members of this dialogue with a constant exchange of views with the project members. They had expressed their appreciation for the process during the internal audit carried out by the project in Q4 2017.

They expressed some concerns about this release, seeking to understand better how various potential risks were assessed. In particular, concerning the "potential impact of the sterile male mosquitoes on the environment" as well as the concerns "that females could be released in addition to the males due to errors in the sex sorting of mosquitoes". The team members had responded various occasions, including by pointing to the risk assessment that the National Biosafety Agency was carrying out as part of the regulatory process that would determine whether or not the release protocol would be receiving approval and be allowed to proceed, and for which the project had submitted data on these specific concerns. Supported by a network of international organizations, these organizations launched a petition and a mobilization to oppose the releases [53]. Following this mobilization, the project consulted the directly affected population to understand if the community shared the concerns raised and if that affected their decision about the proposed release. This was done through a similar process as the original agreement [52], according to the process approved by the institutional research ethics committee and it led to the signature of a new community agreement form. At this occasion and during the public consultation activities carried out by the National Biosafety Agency, and the engagement in the weeks before the release, the community members and their representatives confirmed their agreement for the release to proceed. The two organizations decided to leave the 'relay group' because the exact date of the release had not been communicated early enough to the group. The project had explained that the precise date was dependent on the rainfalls and production cycle of the mosquitoes and, therefore, hard to anticipate. Following this decision, a consultation was carried out with the group's remaining members to understand whether the concerns and criticism on the release date communication were shared and whether specific corrective actions were required. That consultation concluded that the system in place for communication was adequate and that the timing of the release notification—a few days before the release itself—was justified. The group proceeded to nominate a new coordinator and met several times, following the charter rules. The project learned from this experience about the importance of communicating in advance even when there are still lots of uncertainties. Considering the limited time available for participants for these meetings, the project was reluctant to provide information that was still uncertain. This experience showed the importance of sharing information early, even with uncertainties, to strengthen the formal structure for the dialogue with the civil society by demonstrating transparency. This process also highlighted the importance of engaging in regular reviews of the engagement framework to ensure that concerns and questions are adequately addressed.

### Empowerment and accountability

Opening the engagement activities to all stakeholders does not automatically result in an inclusive strategy. Specific provisions need to be made towards the empowerment of the stakeholders in all the groupings. Communities’ empowerment in the research and engagement process requires a certain level of trust on both part, for the communities to engage in a long-term process with the project and agree to share knowledge with the project and from the project to be open to the new demands that this can create, in particular in terms of accountability (Textbox 4).

### Textbox 4: Empowering communities to develop an accountability framework. A case study from the monitoring committee in Burkina Faso

During the preparation phase for the sterile male mosquito release in Burkina Faso, the team worked actively with the village of Bana to establish a monitoring committee. This committee's objective was to have, in addition to the regulatory accountability process that exists in the country, a structure within the village to monitor Target Malaria activities and, in particular, the release process and hold the project accountable. The project proposed a monitoring committee to the community of Bana and worked with the village representatives to codevelop this committee—from its composition to its scope.

The community leaders (chief and his advisors, president and deputy of the village development council) worked with the relevant village representatives (from the village development council, representatives of the different ethnic communities, youth and women representatives) to identify the potential members of the monitoring committee. Criteria were established for the committee to ensure inclusiveness, but also the ability to carry out the task: gender representation, age group representation (ensuring youth would be present), representation of the different village locations, and the presence of at least one person who can read and write to facilitate the reporting. Based on these criteria, the leaders established a provisional list of potential representatives. The project carried out some consultations with a broader group of village members to confirm if the list was appropriate and reflective of the community.

Once established, the village received support from the project to develop its scope and mode of operation. For instance, committee members proposed a list of elements to monitor, and together with the project, modalities for observing them were defined. This included the monitoring of the release itself and the recapture activities and the identification of the collected mosquitoes. Discussions and capacity building activities took place with the committee members to empower them in their monitoring activities and their reporting activities to the rest of the community. This activity led to a meeting organized by the village where the committee members provided a report to the village about what had taken place during the release, and what they had observed during the recapture. The meeting was well attended despite taking place during a busy agricultural season, and the committee members were able to respond to the villagers' questions. They have continued providing information to the village after this meeting based on requests, allowing the inhabitants to hear about the release directly from their observers. This was complemented in early 2021 by formal feedback on the release results from the project to which the monitoring committee took part. This meeting feedback had been postponed several times due to the COVID pandemic and the villagers' desire to do it after the critical agricultural season as they wanted to ensure a large mobilization from community members.

The transparency of the research is an essential component of accountability and empowerment. James et al. recognize the importance of transparency in building trust and carrying out responsible research *"Transparency will be central to trust building"* [21], however, they mainly focus on the aspect of data sharing and do not elaborate on other meaningful ways of being transparent—for instance in phases before the generation of data on contained use or releases of genetically modified strains in the country. Many years before any work with genetically modified mosquitoes took place, the project shared its objectives from the onset and was transparent toward the communities about its research activities. The opening of the containment facilities for visits from community members before operations started was a critical moment in demonstrating this transparency. The majority of community members, including those living near the research centres, had never visited a research laboratory before and admitted harbouring many misconceptions. Community members in Mali even stated that initially, they did not believe that it was possible to breed mosquitoes in a laboratory and, therefore, the visit had been very enlightening. The transparency also entailed making clear that the research project had no guarantee of success and that there are uncertainties that must be answered by further studies and research. This level of transparency created an accountability towards the community members has they were empowered by the information to ask the project about its operations.

The guidance recommendations do not mention the notion of accountability, at the exception of the Guidance on stakeholder engagement for area-wide vector control [33]. Even if that document, accountability is largely restricted to the ethics review committees or regulatory frameworks. In contrast, the project’s response to several recommendation involves an accountability lens, because this is a central value to the project [28]. While accountability was in the project's original ethos [28], an early incident in Mali (Textbox 5) brought even more acute attention to the need to be accountable for its mistakes and be ready to work with communities to recognize them and correct them. The willingness of the project to be accountable and apologize strengthened the relationship with this village, where the project continued to work afterwards.

Textbox 5: Being accountable. A case study from a field entomology incident in Mali In 2014, the field entomology team planned some mosquito collections in the unpopulated area between two villages where it was operating to understand wild mosquito movements better to inform the project's models. As this was an unpopulated area, the team did not anticipate any need for community engagement and agreement before the collection activity. The entomologists set-up at night to collect those mosquitoes, which meant spending the night in this empty area with several researchers and small material, including some lights. They were not aware that the community considered this area a place for spirits by the local community. The next day, local guides collaborating with the project called their focal point in the team explaining that a wave of concerns had spread in the village following the stories from frightened community members who thought they had seen active spirits at night. The project reacted straight away with a visit to community leaders apologizing for the trouble caused and their lack of awareness about the importance of the site for the community and explaining the mosquito collection activity and reassuring the leaders. Following this meeting, the leaders proposed a community-wide meeting and organized to apologize. As a result of this experience, the project changed its field entomology practice and protocols to include the necessity to engage neighbouring communities even when activities occur in unpopulated areas. In addition to this, it raised awareness of the whole team—engagement and field entomology members—about the importance of the local traditions' knowledge and respect.

## Conclusion

When Target Malaria began to establish its engagement strategy, the guidance explicitly addressing the engagement required for area-wide vector control research was scarce. As Fig. 2 shows, the engagement model is not rigid. Instead, it is refined and improved over time through learning from the implementation experience, stakeholder feedback, changes in the context, and recommendations in emerging literature. The phased approach to engagement is part of that dynamic approach as it provides an opportunity to iterate and learn throughout the process.

Thus, the process for learning and self-reflection is critical to ensure that new data—whether from stakeholder feedbacks or new guidance documents—are evaluated and reflected in the strategy if appropriate. The project also created an ethics advisory committee to generate reflection on its approach, activities and way of working. The committee provides an independent perspective on project activities and recommendations to which the project has to respond. The committee meets several times a year, and its agenda reflects both requests for guidance from the project and requests from committee members to explore specific areas of the research activities. These recommendations have been crucial in improving the information process for obtaining individual consent for mosquito collection, for instance. This paper results from the recommendation to better document stakeholder engagement practices and share it with wider audiences who could provide additional reflections and recommendations. On this basis, the stakeholder engagement teams have identified key aspects of their practice and experience that demonstrate the engagement work done over the last years or that addressed key engagement challenges for area-wide vector control research and allocated some time and resources to submit publications to peer-reviewed journals. The team also intends to participate in the broader discussions about the responsible research and engagement considerations for future gene drive development [35] by proposing a practical case to reflect on. While the engagement described in this paper is not applied to a gene drive field evaluation, it is conceived as a pilot phase for learning and building a rigorous engagement model for future gene drive evaluation field releases.

The project acknowledges that it has yet to carry out an external evaluation of its stakeholder engagement activities and strategy. During the early phases of the project, reviews by the various ethics committees and internal audits provided steps towards learning and accountability. However, as the project enters subsequent phases, entailing a broader and more complex engagement approach, the evaluation of stakeholder engagement strategies and activities becomes a priority, not only to comply with existing recommendations [33] but also to align with the project values of excellence, evidence-based decision-making and openness.

This paper shows a specific approach to elaborating and adapting a stakeholder engagement strategy. Because of the project's distinctive nature and the communities that it partners with, this stakeholder engagement strategy is not intended to serve as an example for other projects to replicate. However, it offers a case study to consider community partnerships of other vector control research projects.

In addition, Target Malaria proposes examples of how recommendations can be operationalized, potential challenges therein, gaps in the emerging guidance documents that would benefit from further social sciences research, and an exchange among researchers involved in similar projects of community practices. This responds to comments about the challenges of operationalizing the existing guidance and coming from general principles to concrete implementation methods [35].

While the approach highlighted in this paper was developed in the framework of a gene drive research project, the features are not specifically restricted to a particular characteristic of a genetic approach to vector control. The recommendations from the different guidelines’ documents, though in some cases framed explicitly for gene drive technologies have a broader application that aligns with general best practices for community engagement. Nonetheless, the authors recognized that the development of overall standards in the field of engagement for area-wide vector control (including but not limited to gene drive) would be helpful for external stakeholders to benchmark and evaluate projects’ proposed engagement models and practice.

## Supplementary Information

**Additional file 1.** Methodology. **Table S1.** Review of existing literature and selection criteriaTable of international guidelines for stakeholder engagement.

## Data Availability

The project provides additional data in the supplementary material submitted jointly with this paper.
