# Supplementary material for: Operationalizing stakeholder engagement for gene drive research in malaria elimination in Africa—translating guidance into practice
Source: Malar J. 2022 Jul 23;21:225. doi: 10.1186/s12936-022-04241-3 (PMC9308116; doi:10.1186/s12936-022-04241-3)
Supplement: Supplementary file 1 — Additional file 1. Methodology. Table S1. Review of existing literature and selection criteriaTable of international guidelines for stakeholder engagement. [file 12936_2022_4241_MOESM1_ESM.docx]

Supplementary information: Target Malaria’s response to international guidelines on stakeholder engagement for gene drive research

**Methodology**

Having reviewed a wide variety of publications, the authors have decided to focus on five key publications to demonstrate how the project responds to existing guidelines. The papers were selected along the following criteria: 1) specific to gene drive research for vector control or area-wide vector control research, 2) covering stakeholder engagement aspects broadly and not only the question of consent or community agreement, 3) providing recommendations or guidance to a search in academic publication databases using the keywords “gene drive”, “vector control”, “genetically modified mosquitoes”, and “engagement”. The review excluded papers specifically focused on the molecular biology, entomology or risk assessment aspects as they are not directly related to stakeholder engagement. Table 1 analyses the different sources that were considered and shows how the criteria were applied to inform the selection.

| **Title [shorten, full title in references]** | **Criteria 1: Specific to gene drive research for vector control or area-wide vector control research** | **Criteria 2: Covering stakeholder engagement aspects broadly and not only the question of consent or community agreement** | **Criteria 3: Providing recommendations or guidance** |
| --- | --- | --- | --- |
| **WHO guidance framework**[1] | 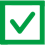 Specific to genetically modified mosquitoes | 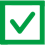 Not exclusively but chapter 4 is specific to ethics and engagement | 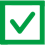 Guidance throughout the text |
| **Gene drive on the horizon** [2] | 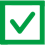 Specific to gene drive | 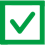 Not exclusively but chapter 7 is specific to engagement | 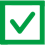 Specific recommendations |
| **Pathway for deployment of gene drive mosquitoes** [3] | 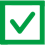 Specific to gene drive | 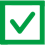 Not exclusively but specific sections on engagement | 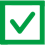 Specific recommendations |
| **Guidance on stakeholder engagement for area-wide vector control** [4] | 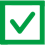 Specific to area-wide vector control research | 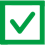 Specific to stakeholder engagement | 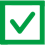 Specific recommendations |
| **WHO guidance ethics and vector-borne diseases** [5] | 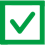 Largely covering area-wide vector control research and sections specific to gene drive | 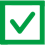 Not exclusively but chapter 10 is specific to engagement | 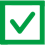 Guidance throughout the text |
| **WHO guidance framework 2^nd^ edition**[6] | 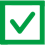 Specific to genetically modified mosquitoes | 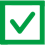 Not exclusively but chapter 4 is specific to ethics and engagement | 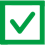 Guidance throughout the text |
| **Knowledge engagement in gene drive research**[7] | 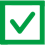 Specific to gene drive | 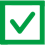 Specific to stakeholder engagement | 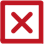 No specific recommendations or guidance |
| **Emerging technologies for invasive species management: the role of engagement**[8] | 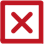 Specific to gene drive but for invasive species | 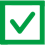 Specific to stakeholder engagement | 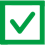 Specific recommendations |
| **Public engagement and communication: who is in charge**[9] | 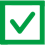 Specific to gene drive | 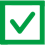 Specific to stakeholder engagement | 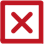 No specific recommendations or guidance on engagement |
| **Recommendation of the CEES** [10] | 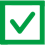 Specific to genetically modified mosquitoes | 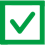 Not specific to stakeholder engagement, but section 4.3 to 4.5 partially address this topic | 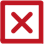 No specific recommendations or guidance on engagement |
| **A typology of community and stakeholder engagement** [11] | 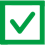 Specific to novel vector control | 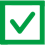 Specific to stakeholder engagement | 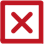 No specific recommendations or guidance on engagement |
| **What makes community engagement effective**[12] | 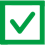 Specific to area-wide vector control | 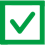 Specific to stakeholder engagement | 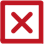 No specific recommendations or guidance on engagement despite highlighting good practices of a specific case study |
| **Articulating free prior informed consent for gene drives** [13] | 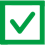 Specific to gene drive | 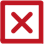 Specific to consent not covering stakeholder engagement | 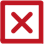 No specific recommendations for practical implementation |
| **Principles for gene drive research** [14] | 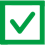 Specific to gene drive | 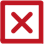 Not specific to stakeholder engagement and only one paragraph addressing the issue | 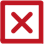 No specific recommendations for practical implementation other than a general call for engagement |
| **A roadmap for gene drives** [15] | 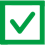 Specific to gene drive | 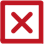 Not specific to stakeholder engagement but several paragraphs addressing the topic | 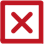 No specific recommendations or guidance |
| **Gene drive for malaria control in Africa** [16] | 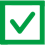 Specific to gene drive | 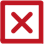 Not specific to stakeholder engagement and only a few mentions | 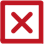 No specific recommendations or guidance other than citing the ones from the NASEM report and the need for early engagement |
| **Discussion paper synthetic gene drives in Australia**[17] | 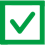 Specific to gene drive | 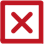 Not specific to stakeholder engagement, only a few mentions | 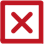 No specific recommendations or guidance on engagement |
| **Informed consent for field trials of gene drive mosquitoes**[18] | 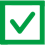 Specific to gene drive | 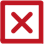 Specific to consent and not covering stakeholder engagement | 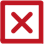 No specific recommendations or guidance on engagement |
| **Informed consent and community engagement for gene drive field research**[19] | 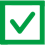 Specific to gene drive | 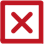 Specific to consent and not covering stakeholder engagement | 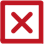 No specific recommendations or guidance on engagement |
| **Gene drive: progress and prospects**[20] | 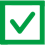 Specific to gene drive | 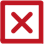 Not covering stakeholder engagement | 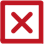 No specific recommendations or guidance on engagement |

Table 1 Review of existing literature and selection criteria

After selecting these key guidance documents, the authors reviewed their recommendations. In some cases[2,4] the recommendations were clearly marked as such, while in other ones[1,3,5,6] they tended to be embedded in the text and needed to be highlighted. Once identified, the authors looked for overlapping recommendations and classified them according to different themes. When considering the integration of the recent second edition of the WHO guidance document on genetically modified mosquitoes[6] (which was published while the paper was in review), the authors look for the new recommendations that were added from the previous edition. The authors recognise that different groupings are possible but choose those as they echo key thematic common to the discussions on stakeholder engagement. The authors then analysed to what extent the stakeholder engagement strategy and activities of Target Malaria respond to those guidelines (Table 2). This was done even for recommendations that were published after some of the activities took place, as a way of reflecting on how the project implemented similar principles in its approach. For each recommendation, the authors propose first an overview of how the recommendation is implemented throughout the project and then one example from one of the three countries. The example is provided to illustrate the response from the project but does not offer a comprehensive image of all the ways the project responds to the recommendation in each country. In other words, the absence of example from one country does not mean that the country team does not apply the recommendation.

**Table of international guidelines for stakeholder engagement**

| **Summary recommendation** | **Original text and reference** | **Project response/example of implementation** |
| --- | --- | --- |
| Recommendations for which the implementation strategy was clear or there were clear precedents from which to learn from | | |
| Being transparent about research work on gene drive technologies, including keeping records of releases (intentional or not) including a description of the investigational product and making them accessible | Thus, transparency should include, but is not necessarily limited to, keeping open and accessible records of any (accidental or intended) releases, containing a full description of the investigational product. [3] | Although the project is still far from field-testing gene drive mosquitoes, Target Malaria is transparent about its research work, and has been publishing its results, even when these results show challenges such as resistance ([Hammond et al, 2017](about:blank)). The project has applied its principle of openness and implemented this recommendation for its first phase of release of sterile male mosquitoes. The project has published on its website a [summary note](about:blank) on the release study, including all relevant information on the strain released. It also intends to publish in peer review journals the results of this release. In addition to this, the project complies with the regulatory process for recording releases and reporting any incident. |
| Researchers are responsible for obtaining fair and legitimate authorization for field-testing gene drive mosquitoes | Researchers are responsible for obtaining fair and legitimate authorization for field-testing gene drive mosquitoes.[3] | Target Malaria is still years away from field-testing gene drive mosquitoes, nonetheless it applies this recommendation to all its activities taking place in the communities, including when working with non genetically modified organisms.  For example, in Mali for the release of wild-type mosquitoes marked by a fluorescent powder (as part of a study to better understand their behaviour), authorization was sought and obtained from two sources. First the institutional ethics committee that reviewed and approved the research protocol, including the process by which the community would be consulted. Second the community, that after a lengthy engagement process involving sharing knowledge, addressing concerns and questions, led to a community acceptance expressed by representatives designated by the community. |
| Engagement activities should take place for baseline studies | It will be essential to begin community engagement and to obtain community support before starting baseline studies. Engagement planning must take into account and be respectful of local cultural protocols. Baseline studies at the planned release site will require the presence of a field team in villages at the study site. The field team should be prepared to represent the project and to describe its goals. Engagement at field sites may involve local community inhabitants, for example, for their knowledge of mosquito breeding sites or as collectors. Researchers should consider how community values, interests and concerns can inform the target product profile. [6] | Target Malaria started its engagement activities when the baseline studies were initiated in 2012. This engagement aimed at providing information about the research objectives and the project’s plans as well as seeking and obtaining individual consent or community agreement prior to any activities. Very early on, the project asked the villages whether they wanted to take part into the field entomology activities to share their knowledge and be part of the collecting team. The feedback shared by the communities through this process is integrated into the project’s thinking about its approach.  For example, in Burkina Faso young people from the villages have been working on collections throughout the baseline studies and were involved in the non gene drive sterile male release protocol that took place in 2019. |
| The process of authorization/consent imply the opportunity to express concerns and to receive replies that address specifically these concerns and to be treated with respect. | Community authorization and informed consent share several key elements. Both promote a deliberative model for addressing ethical issues that arise in connection with research. Rather than relying on strict rules or criteria that must be followed, the deliberative approach mandates that ethical issues be considered before the research is actually undertaken and periodically reviewed. Both are intended as a mechanism for demonstrating respect for persons who will be affected by a research project or a public health intervention. Both imply “voice”, an opportunity to express concerns and to receive replies that are addressed specifically to these concerns. A reply might take the form of assurances or clarification of activities and/or risks, yet for the conditions of voice to be met fully, affected parties must accept the assurances offered as a satisfactory response to concerns. Response also might involve modifications to the plan that relieve concerns, such as additions to RA or RM activities. [1] | During the process of consent for routine field entomology collection, the teams provide information about the protocol, risks, benefits, voluntary participation, etc to the potential participant. During this process, the participant is encouraged to ask questions and the team members address the question or concern with available information in a local language understandable by the individual. At the end of the process, the team asks if the participant has any additional question or requires more time to think about whether or not to give consent. Only after this process, is the consent formally sought and recorded if the participant agrees.  While this process is usually quite short for the routine field entomology activities (one or several meetings depending if it is a new participant, if there are more questions to address or if it is a new collection method), for key milestones involving genetically modified mosquitoes this process can take several months to ensure that all concerns have been expressed and have received adequate responses. For instance, prior to the decision from the community neighbouring the insectary on the importation and contained use of genetically modified sterile male mosquitoes, which complemented the permit provided by regulatory authorities, the engagement with the community took approximately a year. |
| It is advisable to conduct a survey to judge the level of community awareness before asking for endorsement of a release. | It is advisable to conduct a survey to judge the level of community awareness before asking for endorsement of a release. A community liaison or reference group could be helpful for providing feedback on the level of community satisfaction and whether the project is meeting its engagement goals.[3] | In the case of the sterile male mosquito release in Burkina Faso, several assessments were done to evaluate the level of community awareness before asking for a decision about the release. This took the form of surveys asking a sample of community members – including more vulnerable groups – about their understanding of the project and proposed protocol. One was done by the stakeholder engagement team and one was done as part of the project formal audit prior to the release which was conducted by experts from the global team, another African engagement team and an external expert. The results showed a good level of understanding, for instance on the notion of genetic modification, on the release protocol phases, on the aim of the research, etc. The specific details of this study will be part of another publication specific to the preparation of this release. |
| Ethical review of the consent and authorization process must be undertaken periodically. | Engagement is an ongoing process, and community authorization/endorsement must be continually confirmed as new studies are undertaken. Institutional ethics committees, regulators, and community advisory boards likely will play a role in defining the requirements for authorization.[3] | The consent and agreement process for routine field entomology activities are integral part of the field entomology protocols that are submitted and approved by institutional ethics committees. The protocol describes how consent and agreement will be sought and recorded. These approvals are generally for several years but require an annual report to the committee.  In Mali, the institutional ethics committee does an annual review to ensure that the activities are strictly following the protocol. The ethics committee visits one of the participating villages every year (on a rotating basis) and checks that the protocol is being implemented, for instance by interviewing a sample of participants to verify if they sufficiently informed to provide their consent, if they have done so freely and if they have a copy of the information sheet and the consent form.  For the agreement process related to the importation or release of genetically modified mosquitoes, a specific protocol is submitted for approval to the institutional ethics committee, including the information sheet about the release process (with risks and benefits) and the agreement model. The committee provides its approval for the duration of the research protocol.  In Burkina Faso, members of the institutional ethics committee were present for the release of the sterile male mosquitoes in 2019 to ensure that the process was followed according to protocol. |
|  |  |  |
|  | Relevant ethics committees and/or regulatory authorities may need to approve the community authorization plan before release, which should be included in the project development timeline.[3] |  |
| Prior approval from ethics committee(s) should be sought prior to engagement to ensure accountability | Where they exist, projects should seek the approval of relevant institutional ethics committees and/or regional/national committees prior to engagement in order to ensure accountability.[4] | In addition to the protocols for field entomology and importation or release of genetically modified mosquitoes (see section 2), Target Malaria also submits its stakeholder engagement protocol to the institutional ethics committee. This protocol describes the stakeholder engagement strategy and how the team addresses different ethical issues (confidentiality, social justice, respect for persons, etc.). This protocol is then reviewed and only after it is approved by the institutional ethics committee, does the team proceed to stakeholder engagement. The approval is usually provided for several years, but with an annual reporting obligation. |
| It is a standard requirement to obtain ethical clearance for any research involving human participation | At the highest level, safety is a paramount public interest that is addressed through the regulatory mechanisms put in place by governments. Moreover, it is a standard requirement to obtain ethical clearance for any research involving human participation.[3] | The protocols submitted to the institutional ethics committee describe in detail the studies that require human participation and identify instances where individuals are participants to those where individuals might be considered as research subjects. The project does not proceed to any activity involving human participation without ethical approval and appropriate consent or community agreement depending on the type of activity. |
|  | Respect for communities should be an overarching ethical goal in GMM trials. Individuals who satisfy the criteria of “human subjects” must be protected according to Internationally recognized standards.[1] |  |
| Specific budget should be allocated by projects and funders to engagement activities on a long-term commitment | Recommendation 7-3: Funders of gene drive research should allocate a percentage of technical research grants’ budgets to engagement activities, both to encourage good practice and to advance knowledge of effective engagement techniques.[2] | The stakeholder engagement activities have had a specific budget since 2012 and this budget has evolved alongside the development of the activities in partner countries and at global level. Target Malaria’s funders (the Bill & Melinda Gates Foundation and the Open Philanthropy Project) have showed continuous commitment to support those activities.  Budget planning sessions are organized within each team to determine the amount to be allocated according to the results and activities identified in stakeholder engagement strategies during the different phases of the project.  As a point of reference, the stakeholder engagement activities represented approximately 23% of the total Burkina Faso budget for the grant from August 2016 to January 2020. |
|  | In the early stages of the project, a specific portion of the project’s budget should be allocated to engagement work, which will evolve with time as the project moves forward and may differ depending on the context of engagement.[4] |  |
| Funding sources should be transparent to project members and relevant external stakeholders | Projects should ensure that funding sources are fully transparent to both project members and relevant external stakeholders.[4] | The project includes its funding sources in key documents or communication tools (project website, partners website, PowerPoint presentations, factsheets, flyers) and also mentions it when speaking with stakeholders both at the global level and locally. |
| Releases should not be initiated until adequate funds are secure to carry out their regulatory and ethical obligations | If an investigational product meets mutually agreed TPP criteria of safety and efficacy during contained laboratory testing, indicating that it could have a significant impact in reducing malaria transmission in the setting in which release is contemplated, a decision is made to move forward to field testing and releases begin; funders must be prepared to commit sufficient resources to meet long-term obligations to the researchers and to the countries where the testing will take place to which they committed in the research plan.[3] | It is a project decision not to submit a regulatory application for an open field release of genetically modified mosquito strains if the funding has not been secured for the time period of the whole process, including the monitoring activities proposed in the protocol. This decision corresponds to both a risk management measure to facilitate regulatory compliance during the process and to the ethical standards that the project is holding itself to – ensuring that it can comply with its obligations towards the participating communities for the entire duration of the study. As a result, the funding cycles are taking this requirement into account. |
|  | Funders must be prepared to commit to continued support for trial and posttrial activities as long as is required by regulators and by ethical obligations to the community hosting the field testing. Likewise, researchers should not initiate field releases until adequate funds are secured to carry out their regulatory and ethical obligations.[3] |  |
| Independent ethics advisory group can be set up to provide advice, opinions, reflections to the researchers. It can include experts from in-country and involved communities. | They may also wish to consider establishing an independent ethics advisory group to provide additional advice to the project.[4] | Target Malaria set up an independent ethics advisory committee in 2015. This committee membership has evolved with time but includes bioethics, engagement, environmental, religious studies, communication and clinical trial experts from a range of countries, including African countries. They provide advice and recommendations to the project on a variety of subjects related to its operations, plans and ways of working. They receive frequent formal updates from the project about ongoing activities and plans and are also able to ask for specific information to support their deliberations. More information can be found here: https://targetmalaria.org/ethics-advisory-committee/ |
|  | Given the complex ethical and community engagement issues accompanying gene drive technology, an ethics advisory group comprising experts external to the project would be an important mechanism to supplement the input from community advisory boards or other community engagement activities, providing additional and broader perspectives. [3] |  |
|  | Researchers are advised to consider appointing an external ethics committee to advise on sensitive or contentious issues.[3] |  |
|  | Appointing an independent ethics advisory group comprising experts external to the project is recommended.[6] |  |
|  | Mechanisms should be established to enable this group to obtain relevant information on issues such as RA, policy, engagement activities and trial status from the project and other advisors, while ensuring that deliberations within this group remain confidential.[6] |  |
| Other recommendations from stakeholders such as regulators public health agencies, local authorities, and religious authorities, should be taken into account. | In addition, project teams should take into account recommendations from other pertinent actors, such as regulators, public health agencies, local authorities, and religious authorities, even if such recommendations are not legally binding. [4] | The project interacts with a variety of stakeholders whether locally or internationally and is open to their recommendations.  As an example, in 2019, the project has started an engagement at the global level with scholars of religion and local religious authorities to identify key elements in the scriptures and traditions that could inform research process about stakeholders’ value and to engage in an informed dialogue with the project. This process is still ongoing at the time when this paper is being submitted. |
| Engagement plans are needed throughout the research process, including during containment phases, and not only once a tool or methods has been developed | Recommendation 7-1: Research plans to develop gene drives should include a thoughtful engagement plan that considers relevant communities, stakeholders, and publics throughout the process of research, from proposal development through, if applicable, the release and monitoring of gene-drive modified organisms in the environment.[2] | Target Malaria has been committed from early on to engaging stakeholders in the development of the project. The engagement activities started in 2012, when the project established partnerships to explore the feasibility of this technology in Africa. At the time, there was no gene drive mosquitoes for population suppression developed in the lab, but the advancements in the research seemed sufficient to invest in engaging stakeholders to assess whether this technology could be part of the vector control toolkit, and to gather inputs for the development process. In addition to this, Target Malaria has established the same ethical obligations for the communities living around its research facilities in Africa and for the communities where it carries out field work. For example, prior to the importation of the non gene drive sterile male mosquito strain into its containment research facility, the team in Burkina Faso sought and obtained a community agreement from the community living in the vicinity of this laboratory. |
|  | While still working in the laboratory or indoor cage, researchers should consider their obligations to the community in the immediate vicinity of the facility, but should also be planning interactions with the larger public. At this phase, options for the ongoing dissemination of information about the project and discussion with the community could involve establishment of a community liaison group, as well as activities such as “open days” at the research facility where community members can observe the work and speak directly with the researchers.[6] |  |
| Mechanisms need to be in place to foster engagement throughout while avoiding fatigue from stakeholders and recognizing interest and opinion can change and that the process should thus be dynamic | Project teams and stakeholders should discuss and decide together on the mechanisms for engagement and review, amend, update, and refine them as necessary on an ongoing basis. This process requires an initial sharing of information about the work and objectives of the project to help stakeholders understand what is involved. The right balance must be found between early engagement, management of expectations, and stakeholder fatigue. [4] | Target Malaria’s codevelopment approach to engagement activities allows the project to adapt to the changing context and interest of stakeholders. As the project progresses, it gathers feedback from stakeholders about the engagement activities, their form and frequency. This allows the engagement team to adapt its mechanisms to avoid fatigue and take into account the changes.  For example, in Burkina Faso the stakeholders suggested using music, dance and singing for the engagement events to bring some joy and mobilise the community, and this was implemented to mobilise people. As such the team introduced the theatre forum engagement to break the monotony of usual engagement and prevent fatigue. It also seems to have attracted participants who might not have previously been participating to engagement meetings. |
|  | Projects should therefore clarify very early on in the development process the purpose and extent of stakeholder engagement, including stakeholders’ roles, how much flexibility there is for stakeholders’ involvement, and how much teams are willing to modify research processes and activities based on stakeholder input. They should make certain that there is a clear mechanism to communicate how the project team considered stakeholder inputs and the rationale for acting upon them or not. To this end, they should also ensure that the research process remains flexible and dynamic. [4] |  |
| Respectful approach including processes to make the engagement user-friendly and accessible | Projects should therefore allow for sufficient notice and time for a meaningful engagement encounter between project teams and stakeholders in user friendly and accessible venues that break down expert/layperson power dynamics. In addition, they should establish and sustain an institutional attitude and culture committed to respectful engagement. [4] | The co-development approach to engagement processes fulfils this requirement by ensuring that stakeholders are able to adapt the engagement approach to their context and their preferences. The project is also constantly reviewing its activities to ensure that the engagement is accessible and user-friendly. This was for instance the objective of the pilot testing of the use of animated videos in local language to explain entomological collection methods, which aims at complementing the provision of the written information sheets. These animated videos in local language are more accessible than the usual information sheets and their format makes the information session more appealing (based on the feedback generated in the Mali study). |
| Recognition that engagement is a reciprocal exchange of knowledge and that views are not only based on science but also on interests and values | Finally, they should recognise that engagement involves the reciprocal exchange of knowledge between stakeholders and the project team, acknowledging that stakeholders’ and project teams’ views are based not only on science but also on interests and values.[4] | The project has based its approach on the recognition that stakeholders have knowledge, and that engagement needs to be an exchange of knowledges ([Hartley et al. 2019](about:blank)). The project also recognises the importance of values for engagement. For example, based on stakeholder feedback, it appeared that the religious values and beliefs were an important aspect for stakeholders and that they were seeking further guidance on this. The project started an engagement with scholars of religion as well as religious leaders to better understand the principles and values emerging from religious beliefs that could be relevant for this research. |
| Engagement should not be only for instrumental motivations and/or legal requirements. | Instrumental motivations and/or legal requirements should not be the sole basis for engagement. Engagement should be conceived of as a worthwhile pursuit in and of itself beyond the need to satisfy formal requirements.[4] | Stakeholder engagement is one of the three pillars of Target Malaria. Its objectives are not restricted to ensuring consent/agreement for its key activities but rather to ensure that communities can participate in the development of the project, for instance by feeding into the risk assessment process. The engagement is also deeply rooted in ethical principles[21] |
|  | It is crucial that stakeholders and publics invited into engagements of this sort are not made to feel that they are being placated, and that the engagement is simply a stalling tactic with little genuine opportunity for them to have any substantive input[6] |  |
| A community liaison or reference group could be helpful for providing feedback on the level of community satisfaction and whether the project is meeting its engagement goals | A community liaison or reference group could be helpful for providing feedback on the level of community satisfaction and whether the project is meeting its engagement goals.[3] | In various locations, communities decided to put in place a reference group that would liaise with the project, providing feedback but also helping the project to improve its engagement activities by co-developing solutions.  For example, in Mali an activity monitoring committee has been implemented. It is composed of community members and it is monitoring engagement activities and feedback to stakeholders. When something is not well understood, or someone is not adequately engaged, the committee members raise the issue with the project for the team to address this to a satisfactory level for the committee. |
| Contextualization of the engagement is very important. Analysis of the context is also important to elaborate the strategy and ensure that the right tools, mechanisms, timeline and approaches are adopted. | Researchers, funders, and policy makers should adopt engagement plans that are relevant to the social, cultural, and political contexts in which gene drive research may be planned. This contextualization is especially important when the engagement process is organized or sponsored by groups and individuals whose origins and interests are different from those of the stakeholders, communities, or publics to be engaged.[2] | Target Malaria’s core value of co-development addresses the question of the contextualization. The team leading the stakeholder engagement activities are anthropology, sociology, and more broadly social science experts from the country where they operate and as such have a good understanding of that context. In addition to this, they implement international best practices for stakeholder analysis and context analysis to understand what factors need to be taken into account and how to best adapt their strategy. The consultation of stakeholders about engagement approaches is at the heart of the co-development and also participates to this contextualization.  For instance, in Burkina Faso the context has evolved a lot during the period of operation (with social movements, political changes, etc.). Some of these crises have highlighted the strength of the civil society and their importance in policymaking. The project carried out an analysis of the civil society movement, identifying those interested in the questions of malaria elimination, science, innovation and biotechnology. A strategy was put in place to create and maintain a dialogue with those groups, in a collaborative spirit, to ensure that their voices were taken into account, including critical ones. This led to the creation of a “relay group” for civil society organisations. |
| Maximize opportunities for stewardship, ownership, and shared control by the community. | Maximize opportunities for stewardship, ownership, and shared control by the community.[1] | Co-development is a key value for Target Malaria as well as a guiding principle for its engagement strategy. There is a constant attention to identifying mechanisms by which the community can be part of the research process. A large part of this takes place through the co-development of engagement mechanisms, by which the project empowers the community to take an active role in the design and implementation of its activities.  The example of the monitoring committee for the sterile male mosquito release in Burkina Faso illustrate this. The project worked together with the community of Bana where the release was planned to establish a mechanism by which the community members could monitor the release and co-own the process. This committee monitored the implementation of release and whether it was aligned with the explanation given during the engagement process. |
| Role of stakeholders need to be clear for all those involved and the scope for stakeholders’ involvement and adaptation of the research pathway should be made clear. | Projects should therefore clarify very early on in the development process the purpose and extent of stakeholder engagement, including stakeholders’ roles, how much flexibility there is for stakeholders’ involvement, and how much teams are willing to modify research processes and activities based on stakeholder input.[4] | Usually, the process by which stakeholders are involved in project activities is co-developed with the stakeholders giving them control and co-ownership about the scope. In many cases, the project works with stakeholders to formalise those involvements, so expectations are clear from the onset.  For instance, in Burkina Faso the relay group established a charter describing its composition, its way of functioning as well as its role. This charter was signed by all members and by the project team. |
| Plan how the different opinions and perspective will be taken into account in the decision-making processes throughout the research process. | Before releases begin, researchers, in collaboration with government authorities of countries hosting the trial, funders, or other advisors should create a plan for achieving effective engagement with communities and other stakeholders, thereby providing for opinions of various groups to be considered in the decision-making process over the course of a  project. [3] | The project has established clear steps before key milestones where the opinions are perspectives from relevant stakeholders are taken into account in the decision to move forward or not towards that milestone. Those milestones are usually regulatory dossier submission, import and contained use of genetically modified mosquito strains, or the releases of such strain. At those moments, the project carries out an audit to formally seek the opinions and perspective of relevant stakeholders. In addition to these key moments, the project has a more organic approach to integrating the perspectives and feedbacks it receives. For instance, the engagement teams share on a regular basis the concerns they hear from stakeholders and the regulatory and risk team ensure that those are considered when engaging into the risk assessment process. |
| Provide the opportunity for stakeholder inputs at each stage of the engagement activities. The engagement should be adapted to the project development process and timelines to ensure that it is relevant and allows sufficient time for potential changes in project development pathways | The engagement path should be proportional to stakeholders’ understanding of the project, the project’s impact, and the phase of development. Related to this, project teams should have a good grasp of the project development process, including relevant project timelines, in order to ensure that the engagement process is relevant to the broader work of the project and allows sufficient time for potential changes in project development pathways.[4] | The stakeholder engagement activities start well ahead of any key project milestones (usually 18-24 months ahead of a regulatory application for instance) to ensure that there is sufficient time for stakeholders to understand what is being proposed and to feedback to the project their concerns or suggestions. The engagement strategy is based on the various project milestones and therefore offers diverse opportunities for inputs and is a continuous effort throughout the life of the project. As an example, the affected communities are consulted before a regulatory application is submitted, which forms part of the broader readiness check from the project[22]. This kind of consultation includes various opportunities to ask questions, make comments but more broadly to have a dialogue with the project, including requesting for instance to visit project activities in other locations. |
|  | Once a public engagement strategy has been launched, there should be opportunities for follow-up activities. These could include provision for the submission of comments and questions, but might also involve more extended interactions [6] |  |
| Researchers and funders should be open to the possibility that research plans may need to change in response to community input or even that an ongoing project must be halted or moved. | Researchers and funders must be open to the possibility that research plans may need to change in response to community input or even that an ongoing project must be halted or moved. [3] | The project constantly analyses community input to see whether any research plans need to be adapted to minimise the burden on the stakeholders (trying to prevent stakeholder fatigue), or to respond to concerns.  For example, in the process of routine field entomology mosquito collection, the stakeholders in Uganda had expressed some discomfort due to the early morning collection (aimed at capturing mosquitoes while they rest indoor). The stakeholder engagement team relayed this feedback from the affected community to the field entomology team, and as a result the protocol and practice was adapted to reduce this discomfort and collections were scheduled later in the morning. |
| The project should provide feedback to stakeholders about how engagement input was used and how it shaped the project in order to ensure that the engagement process is transparent to all. | Finally, teams should provide feedback to stakeholders about how engagement input was used and how it shaped the project in order to ensure that the engagement process is transparent to all.[4] | The engagement teams work closely with the other functions of the project to integrate stakeholders’ feedback to the activities and pays attention to providing feedback to the stakeholders on how this has then impacted the project’s processes.  For example, the project collected concerns from fishing communities in Lake Victoria about the potential impact on the fish that would feed on modified mosquitoes during aquatic stages. This concern was shared with the risk and regulatory teams of the project and the decision was made to commission a study to assess this and respond to the concern. The study is ongoing and when finalised the results will be shared with the communities as well as with the regulatory authorities. |
| Envisage that non-scientists can enter in a collaborative or problem-solving roles rather than just as subject | Exceedingly complex problems may require planned activities that engage non-scientists in collaborative or problem-solving roles, rather than considering them solely as subjects. [1] | The co-development approach of the project is a direct application of this recommendation. In this context stakeholders are partners of the project and collaborate to find the best processes for the research rather than only be considered as research participants.  The example of the monitoring committee implemented in Burkina Faso (Textbox 4 of the paper) illustrate well this shift to a more active and collaborative role. |
| Involvement of the “end user” (malaria control program, ministry of health) is important to facilitate the public engagement and to understand their plans for vector control campaigns that might impact the research results and sensitize them about the technology and their potential role in its assessment. | Researchers should coordinate engagement efforts with existing regulatory processes and relevant agencies that will be involved in deploying the product. Involvement and input by the end user of the technology, which in the case of gene drive mosquitoes is likely to be the national malaria control program and/or Ministry of Health or equivalent, can substantially facilitate public engagement.[3] | The project has been working closely with the public health sector and more specifically the malaria elimination stakeholders to ensure that their perspective would be taken into account in the development of the technology and our ways of working but also that they could take part in the stakeholder engagement strategy development and activities. At the global level, this is done with the World Health Organisation, in particular with the dialogue with the Vector Control Advisory Group. In African countries of operations this is even more critical.  For instance, in Burkina Faso, the National Malaria Control Programme is a very important stakeholder on two fronts. First of all, it is the focal point for the Ministry of Health as it helps sharing information with the ministry and put this research in the context of the other important efforts taking place in country against malaria. Secondly, the National Malaria Control Programme is participating to the public engagement by sharing the information about this research during the scientific days organised on malaria or other malaria awareness activities. |
|  | Discussion with key opinion leaders should also begin in Phase 1. This would include consultation with government authorities to understand their needs and requirements for a disease control tool, as this might influence product development. This could, for example, include discussions with the national disease control programme.[6] |  |
| Consider the feasibility and appropriateness of engaging with other organisations/programmes to coordinate convergence of messages or collaborations, even in early phases of the research. | Projects should therefore consider the feasibility and appropriateness of engaging with other organisations/programmes to coordinate convergence of messages or collaborations, even in early phases of the research.[4] | Target Malaria is part of a broader ecosystem of organisations working towards one goal: malaria elimination. As part of this work, it has engaged early on in collaborations with other groups to coordinate the public message on malaria research, innovative tools, etc. For instance, the project has a representative in the Strategic Communication Partner Committee of the Roll Back Malaria End Partnership, where it contributes to the elaboration of a global message on malaria.  Similar efforts are done at local level, and for instance the project is exploring collaborations with initiatives such as the Zero Malaria Starts with Me campaign to work on joint engagements in countries where they both operate. |
| Communications should be developed in coordination with appropriate authorities and emphasize information of interest to the community, which might include utilitarian benefit, sustainability, and prudence. | Project communications should be developed in coordination with appropriate authorities and emphasize information of interest to the community, which might include utilitarian benefit, sustainability, and prudence.[3] | The project ensure that the authorities are kept informed about its engagement strategy but also about how it communicates about the research. The team’s understanding of the context and authorities’ perspective helps tailor the communication materials to that context. For instance, in Uganda the project has been closely coordinating with the district health authorities and village health teams when communicating about the results of its activities, integrating their input in this process. More recently, the team has worked closely with the research authorities (UNCST) on the communication about their return to the field after the Covid-19 interruption, to ensure their message could amplify the public health message of the authorities and ensure that the prevention about malaria transmission was also clear. |
| Ensure adequate opportunities and respect for dissenting opinions. | Ensure adequate opportunities and respect for dissenting opinions.[1] | The project has been committed to engaging with critical voices in order to hear their concerns, respond to their potential questions and remain in a respectful dialogue. The project supports the creation of more opportunities for constructive discussions and is committed to cultivating and maintaining core engagement values across all stakeholders.  For instance, at the global level, meetings have taken place with environmental organisations who oppose this research in order to share the project’s perspective and hear their concerns.  Similarly, in Burkina Faso for example, the members of civil society organisations who had expressed concerns about this research were invited to participate to the relay group at the national level that monitors the project’s activities and is engaged on a regular basis. This included proposing visits to the research facilities in an attempt to demonstrate the openness of the project. |
| Plans to evaluate the engagement activities should be in place at every stage of the project and when possible, those should be published or made available. | This should involve making provision for the independent evaluation of engagement activities and, whenever possible, making these evaluations public. Evaluation results can provide feedback and/or demonstrate value propositions to the project teams (e.g., to provide lessons learned to improve/adapt while project activities are ongoing), the stakeholders, the funders, other project partners, and/or the broader network of practitioners and researchers interested in learning about/from engagement.[4] | The project is committed to learning from its activities to refine its approach to engagement. As such, learning reviews are encouraged whether carried out by the team itself or other members of the consortium, or external groups. The project has included a formal audit step in its engagement process, by which a team (mixing experts from the global team, other country teams, and external expert) reviews the implementation of the engagement activities and the readiness of the project to move to a next step. This audit leads to a report, that is so far an internal one aiming at team learning and improvement. However, the project considering publications related to this process, as well as a specific monitoring and evaluation strategy and plan. |
|  | Recommendation 7-5: Researchers, funders, and policy makers should develop and implement plans to evaluate engagement activities related to gene drive research. When possible, these evaluations should be published in the scholarly literature or otherwise made available as part of a shared repository of knowledge.[2] |  |
| Lessons learned should inform the current practice and future engagement plans to improve their effectiveness | Any outcomes and lessons learned relating to stakeholder engagement activities should inform modifications to current practices as well as future engagement plans to improve their effectiveness. [4] | The project is committed to learning from its practice and from the feedback received from stakeholders to improve its engagement and its effectiveness.  For example, in Mali, the team was confronted with some challenges with stakeholders being interested in the project but not being able to attend the group/village meetings as those were conflicting with essential livelihood activities (related to farming). As a result, the team adopted the door-to-door engagement visits, which were proposed by the community members as a suitable alternative. |
| Engagement activities should be accompanied by adequate documentation practices. | All of these activities should be accompanied by adequate documentation practices to record and preserve evaluation information regarding engagement strategies, decisions, and activities, not just for the sake of transparency but also for the sake of informing future project activities and publications. [4] | The project is documenting its engagement activities by recording them on an engagement database, while respecting confidentiality principles. This allows the project to analyse its experience and the stakeholders’ feedbacks but also improve its engagement approach. Similarly, the project continues to reference international best practice for stakeholder engagement and communication monitoring and evaluation tools. In addition to this, in recent years the project has started to dedicate more resources to publish about its engagement activities[23–28] |
| Project should work within the existing accountability mechanisms and a review of existing frameworks (legal, professional codes of conduct, ethical frameworks, documented best practices) should be done regularly to determine whether and how they may be applied to the specific context of gene drive research. | Recommendation 8-10: Research institutions, regulators, and funders should revist international regulatory frameworks, national laws, non-governmental policy, and professional codes of conduct on research and the release of genetically modified organisms to determine whether and how they may be applied to the specific context of gene drive research, particularly with regard to site selection issues, capacity building for responsible and inclusive governance systems, scientific and post release surveillance, and stakeholder engagement. [2] | Target Malaria’s strategy is based on the approach of systematically reviewing existing frameworks as well as new publications to ensure that all best practices, or relevant guidance are integrated into its practice. In addition to the work that is done by the stakeholder engagement team and the regulatory affairs team, this work is also done by the ethics advisory committee on a regular basis. |
|  | Projects should also work within the existing accountability mechanisms, such as national and international legal frameworks, research ethics protocols, and human rights principles.[4] |  |
| Engagement requires a multi/inter-disciplinary approach including experts with knowledge of the local context. | An interdisciplinary team should include a dedicated engagement group with appropriate skills. The particular set of skills deemed appropriate not only will depend on the specific investigational product, context, organisation, and objectives of the team but may also include mediation, facilitation, multiparty consensus building, social work, community mobilisation, communications, youth outreach, conflict resolution, public relations, etc. Teams should include experts on a diverse range of topics, including local culture, economics, politics, and history. [4] | The co-development value of the project also shapes how the engagement expertise is developed internally. The engagement team is composed of researchers in social sciences but also engagement practitioners with field experience on how to engage communities. Those teams bring the engagement expertise, but they work very closely with the field entomologist and other biologists of the laboratory to elaborate the engagement strategy and develop the appropriate communication about the project activities. This work is also supported by the communication experts who support the development of tools and channels to facilitate this dialogue with stakeholders.  In each country of operations, Target Malaria works with a national research institute or university and all the engagement staff (as well as staff operating in other fields) is from the country or region. They all demonstrate excellent knowledge of the local context.  In addition to this, for instance in Mali, the project recruited members of the villages to be part of its stakeholder engagement team. The idea was that those members would have a good understanding of the local dynamics, traditions, and would provide an extra later of local knowledge to the rest of the engagement experts of the team. |
|  | Because the nature of effective engagement is so context specific, it is best undertaken by people who are locally known and respected and have deep knowledge and understanding of the local value system and culture.[3] |  |
|  | The composition of the research team should reflect the process of engaging with local communities, gathering this information and integrating it into the project’s planning and deliberation process. Depending on the competencies of both project staff and locally affected parties, it may be appropriate to include representatives from affected groups within the project’s governance mechanisms.[6] |  |
| Scientists and researchers should have a clear understanding about their role in engagement and should be able to provide accurate information about the project and its goals to stakeholders when required. | Such a team should also ensure that scientists and researchers have a clear and explicit role in engagement activities and a clear understanding of the rationale behind them. They should provide stakeholder engagement experts with support related to the technical and scientific aspects of the project and be actively involved in discussions with stakeholders in order to address technical questions and concerns and ensure direct two-way communication between project teams and stakeholders.[4] | While there is a stakeholder engagement team dedicated to this activity, the other scientists and researchers also play a key role in engagement. They can for instance support the engagement team on the development of clear explanations to make the complex details of the science accessible for different publics. They also play an important role in some engagements where stakeholders want to hear directly from the researchers. The roles of each team are clearly stated in project internal document, and coordination takes place on a regular basis to ensure that everyone is aligned.  For example, in Burkina Faso, the science team has played a key role in helping stakeholders understand the concept of containment and of mosquito rearing and research. The stakeholder engagement team organised the activities (insectary visits, open days, etc.) and facilitated the events, but the scientists from the insectary explained the different features and responded to stakeholder questions. |
| Specific skills will be required to develop the necessary vocabulary to accurately and understandably convey the technical aspects of the research. | Researchers should include experienced science communicators on their team, as well as sociologists and linguists to help develop the necessary vocabulary to accurately and understandably convey the technical aspects of the research to each group of stakeholders. [3] | A number of technical terms or concepts are required to explain the different phases and activities of Target Malaria. Those terms and concepts might not be very accessible, and their translation to another language – in particular local dialects where scientific terminology is not formalized – might add an extra challenge. The project supported in each country a research work with the communities to co-develop a joint terminology that could be used to talk about those aspects.  The details of this work can be found in Chemonges et al. 2021[25] |
| Opportunities for communities to meet with project leadership if desired should be available. | Although this is best performed by project team members who are social scientists familiar with the local culture and are experts in engaging community members, there should be opportunities for the community to meet with project leadership, if desired.[3] | The project dedicates significant time and resources to maintain a close relationship between its leadership in country and the communities. Principal Investigators of the various partners participate to stakeholder engagement activities, including insectary visits, questions and answers sessions, etc.  For example, in Burkina Faso, on an annual basis the leaders of the community and various stakeholder groups are meeting with the project leadership who is thanking them for their partnership but also taking the opportunity to have a dialogue session. In between those sessions, it is not rare for the PI to participate to engagement activities, whether locally or at national level, and to be accessible for stakeholders to directly interact with him. |
| Support and/or formal training in conflict management and in communication for relevant personnel. | Relevant study personnel should receive support and/or formal training in conflict management and in communications.[3] | Target Malaria recognises the importance of addressing some challenges with the appropriate broad range of skills needed for the diverse activities and operating conditions within the project and as such organises appropriatedequate training for its personal. Communication trainings have been organised for partners in Africa as well as Europe, and refresher trainings are taking place on a regular basis. To this date, no formal training in conflict management hasve taken place as the teams have so far felt sufficiently equipped, but this recommendation will be further discussed within the project |
| Projects should ensure that their teams are sufficiently informed to take part actively in dialogues with stakeholders. | To achieve this, projects should ensure that teams are sufficiently informed so that they feel they have the capacity to take part actively in dialogues with stakeholders. [4] | The project invests resources and time for internal communication and training to ensure that all team members can provide accurate information about the project. Tools such as an internal newsletter, communication training modules, key message maps, regular working group on specific topic are in place to achieve this. The project is currently working on e-learning modules on engagement and communication to foster this knowledge sharing in a time of travel constraints. During audits, the audit team verifies the ability of project team members to explain the project and its activities in a meaningful way to stakeholders. For instance, before the release of the sterile male mosquitoes, the audit checked that team members were able to explain the protocol, including the monitoring activities. |
| Stakeholders can be empowered to become knowledge sharers themselves, but the responsibility of transmitting information remains with the project. | Ideally, information sessions should equip stakeholders to become effective knowledge sharers themselves, able to accurately communicate the most salient information about the project to their peers and others in their spheres of influence while ensuring that the responsibility of transmitting information does not fall too heavily on actors external to the project.[4] | Target Malaria is trying to empower different individuals or groups to share knowledge about the project.  For example, in Uganda, the team has identified individuals within the field sites and the community around the insectary (local champions or peers) to support efforts to reach out to the hard-to-reach stakeholders and share basic knowledge about the project (project objectives and some activities). There is also a close working relationship with the Village Health Teams, teachers and religious leaders to share knowledge on malaria and the project. These champions are biannually trained on the basic information about the project that they can easily share with other stakeholders. However, for the more detailed and complex information about the project and the science, this is the responsibility of the designated engagement teams. |
| Allow stakeholders to physically visit project facilities or participate in some activities. | Relatedly, projects should consider allowing stakeholders to physically visit project facilities or participate in some activities in order to foster knowledge of, and familiarity with, the activities of the teams.[4] | The project organizes visits for stakeholder groups to the insectary to increase their knowledge of the activities of the insectary. These visits are important for the project because they build the knowledge and confidence of the stakeholders for the research activities undertaken in a contained laboratory.  Since the inauguration of the insectary in Uganda in July 2019, the project organized visits for the stakeholders around the insectary to build their knowledge of the insectary work In August and September 2019. During the same period the project also organized a visit to the insectary for members of the civil society. |
| The project should update stakeholders of any change or new development | Finally, projects should ensure that stakeholders have a good grasp of the scientific development process, including relevant project timelines, and should make certain to update stakeholders and project teams with new information from both the project and the community as the situation evolves.[4] | Target Malaria shares relevant information about changes of strategy or plans, in line with its value of openness and accountability. In addition to this, the project is very clear about the uncertainties in its processes, and the fact that changes are inevitable.  For example, in Uganda, there was a recent change of field sites selected, due to a change in entomological strategy. Once this decision was made, all the relevant stakeholders were notified such as regulators (Uganda National Council for Science and Technology) and other stakeholders within the Ministry of Health. Similar information and dialogue took at the districts and field/village sites affected by the decision of change of sites. This included members of the community who participated to the study as well as the broader population. |
| Trials should be clearly described to the community members with authentic explanation of risks and benefits, and seek their perspectives and the project should seek their perspectives. | The engagement team should clearly describe the trial to the community members, with authentic explanation of risks and benefits, and seek their perspectives. [3] | Target Malaria has done yet started trials with gene drive mosquitoes and those are still a few years away. However, it is committed to sharing openly the design of its studies with the community members including potential risks and benefits and to consider communities’ perspectives in the process.  For example, in the case of the sterile male mosquito releases in Burkina Faso in July 2019, the project took time to describe the mosquito strain and the protocol to the community as well as discuss the potential risks and benefits of this process. The concerns and perspectives of stakeholders were addressed but also shared with the other team members as well as the experts carrying out the risk assessment (both internal and independent ones). This whole process was submitted to the institutional ethics committee of IRSS prior to its implementation to ensure its ethical appropriateness. |
| Stakeholders should be informed well in advance of the modalities and rationale for any eventual cessation of project activities as well as exit from local communities | Finally, stakeholders should be informed well in advance of the modalities and rationale for any eventual cessation of project activities as well as exit from local communities. [4] | When a decision to cease activities at a field site/s is made, Target Malaria informs the stakeholders about the decision and the rationale and encourages dialogue with the communities in line with the openness and accountability values.  For example, in Uganda, in 2017 the project decided to cease activities at two Island sites in Mukono district due to entomological reasons. Before communicating the exit, the project team met and discussed the process of exit management. A message for the exit and communication tools were carried along to inform the stakeholders at the district, sub-county and the local communities in the two island sites. This exit process was done with time to allow a dialogue with stakeholders, ensuring that the rationale was clear but also providing the time for the project to thank the community for its support in the research. When such exits take place, the project ensures that the message on malaria prevention is clearly passed so communities can continue protecting themselves from malaria using conventional tools. |
| Engagement with media is important to foster information. | Fostering well-informed media is an important consideration throughout all facets of product development and testing. It will be important to engage proactively with the media, for example, by offering accurate, fair, and balanced informational sessions and tours of the research facilities. This will help the media to obtain a basic understanding of the project and to provide accurate material and information about gene drive technology.[3] | Media engagement is an important component of the project. Since 2018, a communication function was put in place in the three partner countries to foster this collaboration with media in country. A media engagement strategy was designed in the following period to accompany the stakeholder engagement strategy and allow the project to reach a broader public.  For example, in Uganda, in July 2019 Target Malaria conducted a series of activities involving the media to support the inauguration the new insectary (Arthropod containment level 2) at the Uganda Virus Research Institute. Prior to the inauguration the insectary the stakeholder engagement and the communication team identified key media based on their expertise in terms of reporting health and malaria related stories. The project team visited the media houses to brief them about the project and also officially invite them for the inauguration event. On the inauguration day, the project team organised a press conference for the journalists to interact with the researchers and also provided the journalists with information materials which included press release and factsheets. |
| Researchers should identify project spokespeople and provide them with communications skills that will enable them to explain the project clearly to stakeholders and the media | Researchers should identify project spokespeople and provide them with communications skills that will enable them to explain the project clearly to stakeholders and the media.[3] | Target Malaria has specific staff who have been appointed to speak on behalf of the project. All those spokespeople have received media training to support them in this task and help them explain the project clearly, trying to avoid jargon. Frequent refresher trainings are taking place to ensure that even if they are not using this skill very often, they are still equipped to be a spokesperson. In addition to this, the communication team provides support to these spokespeople before important speaking events. |
| Project should be ready to work with the community to respond to its needs in the case of a malaria outbreak during the period of a trial. | Researchers must be prepared to work with the community and respond to its needs. For example, this may involve temporarily halting releases or ensuring malaria treatment is available in the area where malaria has appeared.[3] | The countries where the project operates are highly endemic countries and therefore most National Malaria Control Programmes have plans in place in the case of exceptional malaria prevalence. The project would open a dialogue during the trial design phase with the health authorities, the National Malaria Control Programme and the communities where the study takes place to identify if additional response plans should be prepared. |
| Stakeholders should be consulted and participate in designing the grievance and complaint mechanisms. | Stakeholders should be consulted and participate in designing the grievance and complaint mechanisms to ensure that they meet their needs.[4] | Stakeholders were consulted in the implementation of the complaints management mechanism. This consultation allowed researchers to integrate stakeholder inputs so that the mechanism could take local realities into account.  For example, in Mali, community members shared how issues are resolved in their village and proposed to incorporate this in the way the grievance management system of the project would function. As such, the project has integrated the chief and his advisors into the process, while also allowing for other routes to express grievances if community members wish to do so. |
| Challenging recommendations where tensions were identified, and the implementation strategy was more ambiguous/requiring more work | | |
| Acknowledging potentially challenging issues [including uncertainty of outcomes and risks] and navigating them with openness to learning | Projects should avoid omitting problematic topics or themes, including uncertainty of outcomes and risks. This will involve proactively acknowledging potentially challenging issues and navigating them with an openness to learning rather than defensiveness as well as recognising that the need for transparency in information sharing also encompasses information about lack of success or delays. [4] | In its engagement Target Malaria makes clear to stakeholders that it is a research project and highlights the uncertainties that are associated with its work, including the uncertainties about the technology being effective, or the associated risks. When responding to questions about aspects of the research that may develop in the future, the project reaffirms its values and shares its pathway to respond to the question in an open way.  For example, in Burkina Faso, when it initiated its engagement, the team spent time explaining the difference between a research project and a malaria intervention and the associated uncertainties. |
| Household consent may also not be appropriate when more than one mentally competent and autonomous adult or family shares a household and no single adult person is deemed to be a representative of the household. In such instances, one adult may give household consent on behalf of other mentally competent adults in that household who hold different views on the provision of consent. | However, the notion of household consent may be problematic because of the implicit power dynamics within a household. For example, consent on behalf of the household is often given or refused by the male head of the household, which may reflect a patriarchal bias. Household consent may also not be appropriate when more than one mentally competent and autonomous adult or family shares a household and no single adult person is deemed to be a representative of the household. In such instances, one adult may give household consent on behalf of other mentally competent adults in that household who hold different views on the provision of consent.[5] | The project operates in some villages where the concept of household is not culturally appropriate as the unit of decision-making. For instance, in Mali and Burkina Faso where families are usually organised in a compound with several households gathered around the same courtyard, the traditional decision-making mechanisms are different and therefore the consent process has been adapted to this (by the project but more generally in research taking place in those settings). Usually the head of the compound, who can also be the head of the family (family is here a broader unit than the nuclear family of parents and their direct children), provides the consent for activities taking place in the compound. In addition to this, the occupant of the room that is being sampled confirms his/her consent for the activity. In Uganda, the village is organised by household, so the designated head of the household provides the consent. |
| Stakeholders might be engaged at different level according to the level of impact of the project. These differences should be made clear | They should distinguish to what extent stakeholders with different levels of involvement are consulted. Communities directly affected by the project will need to provide their acceptance of project activities that will have immediate impacts on them and approve the evaluation for the research to progress, whereas other stakeholders who have an interest in the use of the intervention or knowledge that could contribute to the intervention’s development and potential use need to be engaged, but their acceptance may not need to be formal. Finally, the public at large (for instance, in nonendemic countries) needs to be engaged but may not necessarily need to accept the evaluation or use of  an intervention. [4] | The project’s engagement strategy is focused on prioritising the most affected populations. As it progresses to next phases, stakeholders who are the most affected will change. The project communicates clearly about its focus on the most affected populations and explains clearly to other stakeholders the difference in the level of interaction, engagement as well as requirement to provide an agreement for the activities[26].  For example, in Burkina Faso, during the engagement activities prior to the release of sterile male mosquitoes, the community of the release site was prioritised as the one directly affected by the activity. Other stakeholders (for instance at national level) were informed and engaged to gather their concerns and expectations, but it was always made clear to them that only the community of the release site would be providing an agreement for the activity to proceed. |
|  | The project team must develop and implement planned activities to consider the interests of these third parties and engage with them in a respectful manner[6] |  |
| The engagement mechanism should be discussed and decided upon together with the stakeholders, and can be reviewed on an ongoing basis as necessary. | Project teams and stakeholders should discuss and decide together on the mechanisms for engagement and review, amend, update, and refine them as necessary on an ongoing basis.[4] | Based on its co-development principle, the project believes that the engagement activities and mechanisms need to be developed and agreed upon with the stakeholders. Frequent reviews take place to ensure those are still valid. Reviews or adaptation of engagement mechanisms can be initiated because of stakeholder feedback, because the team enters in a new period of work and is reviewing its approach, or because of lessons learned from experience. |
| The mechanisms should take into account the power dynamics and imbalances and provide mechanisms to ensure that a diversity of voices can be heard. | These mechanisms should take into account intracommunal power dynamics and imbalances so that representatives of a wide range of demographic and identity groupings feel able to engage actively.[4] | In accordance with its principle of focusing on the people directly affected and on the vulnerable groups, the project pays particular attention to adapt its approach to ensure social justice in its processes and ensure that all voices can be heard. This is done thanks to the frequent stakeholder analysis made by the team and the strategy based on this analysis to ensure that the engagement is inclusive despite potential power dynamics and imbalances.  For instance, in the villages of Burkina Faso where the team operates, there are a variety of ethnic groups present. The villager founders’ group of bobos have the uncontested village leadership and as such make the key decisions, while consulting the various constituencies of the village. Because of the anthropological study done to analyse the village, the strategy is based on different layers of engagement, with activities specifically targeted at the groups that are not part of the village founders to hear their perspective and ensure that it has been represented in the general dialogue between the village and the project so it can be taken into account. The approach consisted in checking with each subgroup, in particular more vulnerable groups, whether the engagement activities were appropriate for them and whether they felt represented by the group taking decisions on behalf of the village on the project. |
| Potential training might be needed for institutional and national ethics review committees related to this research as they often lack vector control expertise and awareness of ethical issues in entomological research | Additionally, there may be a need to train institutional and national ethics review committees on the importance and process of ethical review of GMM trials. In both developing and developed countries, ethics review committees often lack vector biologists and awareness of ethical issues in entomological research protocols/proposals. Attempts should, therefore, be made to create awareness of such issues among committee members responsible for approving and providing oversight for the planned trials, and to encourage the committees to seek appropriate expertise when considering GMM research/trials.[1] | The committees, whether institutional or national are considered as project stakeholders and as such they are engaged through different activities to share knowledge with them about the project activities, the science behind the research, and to have a dialogue about their potential questions and/or concerns. This is largely informal but, in some instances, (for instance in Mali during the period of review of the application for importation and contained use of sterile male mosquitoes) the project has been asked to come and formally present to the national ethics committee.  Notwithstanding these meetings, the project would support other actors who are not involved directly with the research to provide additional trainings (for instance national malaria control programmes, WHO, etc.) |
| Recommendations vague in nature and requiring a significant interpretation according to the project’s values, principles and operating context | | |
| Because the nature of community authorization will vary in different contexts, it is essential to investigate what the community itself considers to constitute a valid authorization. | Because the nature of community authorization will vary in different contexts, it is essential to investigate what the community itself considers to constitute a valid authorization. Therefore, identification of key stakeholders and community representatives is vital, and early and ongoing engagement with these parties is essential. [5] | The project has leveraged its routine field entomology activities to develop and test models of community agreement. For instance, the project collects mosquitoes in swarms which are taking place in the common outdoor living space, thus community agreement was the adequate form of consent. The project investigates how the community wanted to provide its decision on whether or not to allow this activity. This investigation takes place in each village/location as this can vary even within a country. But the process is similar, usually the legitimate leadership of the area is asked what the process for community decision is and the project also verifies with the rest of the community (asking different groups, incl. vulnerable ones) that this process is appropriate and legitimate.  For example, in Mali the project explains the protocol of the activity to the leadership of the village and addresses their questions/concerns and reiterates this process with a broader group where key constituencies are represented (youth, women, etc.). After this, the village carries its own consultation process based on traditional practices for a minimum of three days and then provides the decision to the project. |
| For activities requiring consent, similar requirements to research involving human subjects should be applied | Epidemiological efficacy testing necessarily  will involve interactions with human participants living in the trial area for the purpose of collecting individually identifiable information and/or specimens, and this must be conducted according to standards for human subjects research.[3] | For research involving human subjects, consent is ontologically individual, voluntary and is acquired through a transparent, free and informed decision-making process. The person has the option of whether or not to accept the research and of withdrawing from the study at any time without any prejudice.  In Mali, the project required individual consent for room owners and occupants before carrying out a spray catch mosquito collection. This consent process is the subject of a protocol submitted and approved by the institutional ethics committee, including the information sheet that ensures that the participant is aware of risk and benefits, the research objectives as well as his/her rights during this process. |
|  | Appropriate ethical clearance is necessary for studies involving human participation. At any stage of testing, informed consent is required for those who meet the internationally accepted criteria of research subjects[3] |  |
| The identification of the people impacted will vary according to the protocol design and should be taken into considerations in the risk assessment process. | The distinction between people who are affected directly by research and others who are more indirectly interested in its conduct may be operationalized in the way that the relevant ethical obligations are understood. For example, when research involves risks associated with organisms or substances released into the environment, as opposed to contained within experimental facilities, geographical proximity to the site of research becomes an important ethical indicator. In the case of some GMM trials, defining the limits of potential effects may be complicated by the geographical mobility of both people and mosquitoes’ overtime. Such considerations should have been taken into account in a RA, which will be helpful in guiding identification of community stakeholders.[1] | The various functions of Target Malaria work closely together during the protocol design phase. This allows the project to ensure that stakeholders’ inputs or constraints are taken into account but also that the engagement team can provide insights for the protocol design. The engagement team also works closely with the risk and regulatory affairs functions to provide information about the affected people.  In addition to this, Target Malaria will be commissioning an Environmental, Socio-Economic and Health Impact Assessment for the next phases of releases. This assessment follows a structured process to identify impacted people and to take into consideration their inputs for the impact management plans. |
|  | During small-scale field testing, it is important also to include concerns expressed by community members at the trial site and by involved third parties, such as government officials, in the risk assessment process.[3] |  |
| The engagement should expand to national, regional/multinational levels depending on each phase of testing. | Because of the potential for geographic spread of gene drive mosquitoes, engagement must expand rapidly to the national and multinational levels (as addressed for each testing phase in the following text).[3] | The Target Malaria engagement strategy has always included a multi-level approach, while prioritising directly affected populations. As the project is moving towards the release of gene drive mosquitoes, which will persist for longer in the environment and thus could spread geographically, the need for regional and multi-national levels engagement is increasing. The project is taking the opportunities of existing regional for a (for instance workshops and dialogues organised by NEPAD/AUDA) to engage at a broader level. |
| Projects should consider supporting activities that enable stakeholders to deliberate and express their collective voices | Projects should consider supporting activities that enable stakeholders to deliberate and express their collective voices. [4] | The project considers supporting activities on a case-by-case basis to facilitate deliberation and empowerment of the stakeholders to express their collective voices  For example, in Burkina Faso, the group relay has made request to get more capacity and training to better understand the technology and underlying knowledge, and the project is exploring how to support that. |
| Coordination of the engagement efforts with existing regulatory processes and relevant agencies that will be involved in deploying the product. | Researchers should coordinate engagement efforts with existing regulatory processes and relevant agencies that will be involved in deploying the product.[3] | A potential deployment of this technology would require a close coordination with the regulatory agencies and the public health authorities including for the engagement process. The deployment of this technology is still many years away and the project has adopted a gradual strategy to discussing this aspect with various stakeholders that reflects the stepwise approach to the research. Nevertheless, the teams are working closely with those groups to share their stakeholder engagement approach and integrate learnings or feedback from those agencies and experts. When the project meets them, the presentations usually include a section on stakeholder engagement, to provide an overview of the strategy as well as any achievements or challenges. |
| Dissent should be captured and considered carefully, but engagement does not require the dissenters to be convincing or convinced | Moreover, engagement mechanisms should make space for a diversity of opinions, including those that may be contrary to the views of projects, without the necessity for dissenting voices to be convinced.[4] | The stakeholder engagement teams record systematically the concerns from stakeholders, including dissenting voices, and according to the process set in the protocol approved by the institutional ethics committee. This is also shared with the scientific team and the regulatory affairs team of the project to ensure that those get considered during the risk analysis and product development.  In the case of Burkina Faso when some dissenting voices decided to leave the relay group because of their disagreement with the project’s upcoming release at the time, the project respected the decision, and engaged the rest of the group to see if anything could be learned from this decision and improved in the process. |
|  | Recommendation 7-8: Researchers, research institutions, and other organizers should design engagement activities to respect different points of view. Such deliberation may enable participants to reflect upon their own beliefs and understandings in new ways. Dissent should be captured and considered carefully, but engagement does not require the dissenters to be convincing or convinced.[2] |  |
| Information and communication materials should be tailored to the audience, their interests and concerns as well as translated to appropriate language(s). | Good communications materials, translated into the appropriate language(s), will be vital for explaining the technology and, therefore, will underpin engagement efforts at all levels.[3] | Information and communication materials have been developed by the project to support engagement and communication with stakeholders at different levels (from village to global through national).  For example, the project has developed specific power point presentations, factsheets and short videos for communication about the project and its science with the stakeholders at the national and global level. Whereas for the stakeholders at the field/village sites, the project has developed tools that are more visual taking into consideration education levels as well as scientific knowledge. The project is either using materials directly in local language (as it is the case for videos on entomological collection) or is using local languages in engagement activities (e.g., for the theatre play in Mali). The project is also using the feedback from stakeholders to prepare new communication materials responding to concerns or expectations. |
| Identify opportunities for individuals/households at the release site not to participate and what those opportunities might comprise and involved advisors and communities to map those opportunities ensure community members understand how to access and use them. | An important consideration is whether opportunities can be made available for individuals or households at the release site to choose to not participate and, if so, what these opportunities might comprise. Researchers should remember that there will be a need for engagement around these options, to ensure that community members understand how to access and use them and what they offer in the context of gene drive.[3] | Target Malaria is committed to showing respect to all community members, including those who might not want to participate in its activities. The project has not yet been faced with a specific example of individuals or households expressing their desire not to participate in a genetically modified mosquito release. However, this is a clear area identified for further work as highlighted in Thizy et al. 2021[26].  The closest experience with this is the one related to study participants who decide to withdraw their consent. While those are relatively rare cases, they are closely analysed by the project, to ensure that those who decide to leave the entomological collection study are respected in their choice by the project and the community. The annual report to the institutional ethics committee includes a review of those cases – while maintaining their confidentiality. |
| Assurance that the community has access to the standard of care for malaria according to national policy during gene drive trials, which will vary according to the experimental strategy and could require coordination with the health care system. | Researchers should anticipate that ethics committees and/or regulatory authorities will require assurance that the community has access to the standard of care for malaria according to national policy. Obligations may differ according to the experimental strategy, for example, if female mosquitoes will be released. Access to long-lasting insecticidal nets is required at this stage if not previously provided during baseline studies, because this represents the current best practice. Access to malaria medication is not usually a research project’s responsibility, but it is recommended that researchers work with the health care system to ensure that it is readily available.[3] | The project is still years away from any release of gene drive mosquitoes. However, it is already starting to coordinate with the National Malaria Control Programmes in the country where it operates. As for the evaluation of other vector control strategies, the field studies of the project take place in areas that are under the jurisdiction of the National Malaria Control Programme and the bednet distributions and replacements as well as other measures (such as indoor residual spraying) are not affected by the protocols implemented by the project.  In a different context, the project is already implementing such coordination with the public health systems to provide standard of care for malaria when carrying out human landing catch mosquito collections. |
| Malaria management strategies will be needed as part of the trial design and should be discussed with the community in advance. | It is possible that a malaria outbreak will occur naturally during testing or follow-up, for example, as a result of rains that support mosquito development. This possibility, along with anticipated malaria management strategies, should be discussed with the community in advance. Such an outbreak can be expected to trigger a need for intensive community engagement and broader public communication efforts.[3] | The field trial design for gene drive studies is still in its early phase but this recommendation will be considered carefully and discussed with the communities as well as the health authorities. |
| Engagement should be integrated in the construction of risk assessment models and those should be feedback to communities. | Recommendation 7-2: Because engagement can contribute to defining the values and preferences of communities, stakeholders, and publics about gene drive technologies, researchers and risk assessors should integrate engagement into the construction of risk assessment models. In turn, the outputs of risk assessments should feed back into engagement efforts.[2] | The project takes communities’ feedback very seriously and their comments have been integrated to the risk assessment processes. The concerns and expectations from sterile male mosquitoes were for instance shared with the Commonwealth Scientific and Industrial Research Organisation (and Australian expert group specialised in environmental risk assessment) which was carrying out an independent risk assessment of the release of this strain. The experts looked at the concerns to identify whether there were any plausible pathways to harm that needed to be integrated into their analysis.  The project shares with the community the process and results of these risks assessments, in particular in response to their key concerns. |
| The results of the risk assessment should be made publicly available in the interests of transparency, and to facilitate trust-building between the research team and the community. | Before moving from one level of testing to the next, researchers or funders should commission an external all-hazards risk assessment; this will inform project planning and decision-making, and also build trust if made publicly available. [3] | The project has made its risk assessments publicly available in two ways. First by publishing those reports on its [website](https://targetmalaria.org/wp-content/uploads/target-malaria-independent-ecological-risk-assessement-small-scale-release-sterile-male-executive-summary.pdf) (e.g., with the independent risk assessment report from CSIRO[29]), but secondly by having more meaningful engagement about this with the communities where the risk assessment reports might not be accessible in their usual form (due to their technical aspect for instance). |
|  | The results of the risk assessment should be made publicly available in the interests of transparency, and to facilitate trust-building between the research team and the community.[5] |  |
| Risk of engagement should be identified and appropriately mitigated. | Stakeholder engagement activities often entail risks to stakeholders and project members. Project teams should give appropriate consideration to the means of identifying and mitigating them. [4] | As part of their stakeholder engagement strategic thinking, the teams identify the risks of engagement and establish a mitigation strategy. For instance, the risk of stakeholder fatigue and the risk of engagement activities conflicting with essential livelihood activities during key seasons (such as harvest) were identified and the engagement teams have been working with the communities to see how to best adapt those activities and strategy to mitigate those risks and minimise the potential impact of the project on the communities. |
| Project teams should establish clear and accessible grievance and complaint mechanisms and inform stakeholders of how they function | Project teams should establish clear and accessible grievance and complaint mechanisms and inform stakeholders of how they function.[4] | The project has established a complaint mechanism at each one of its field sites and around its research facility. Communication materials (such as posters) were developed to ensure that community members were aware of this process and able to use it. The project is currently working on how to upscale these mechanisms to ensure that any relevant stakeholder in the country in which it operates who wants to express a valid concern, or a complaint can do so and be heard by the project. The question of accessibility of the process was at the heart of its design to ensure that literacy level, location, or other constraints would not be an obstacle to express a grievance.  For instance, in Burkina Faso, posters with images and text in local language were put in all villages where the project operates to explain how the grievance mechanism works, in addition to the information sessions organised with the communities to explain the process. |

1. WHO/TDR and FNIH. Guidance Framework for testing genetically modified mosquitoes. 2014;159.

2. National Academies of Sciences Engineering and Medicine. Gene Drives on the Horizon. 2016.

3. James S, Collins FH, Welkhoff PA, Emerson C, Godfray HCJ, Gottlieb M, et al. Pathway to Deployment of Gene Drive Mosquitoes as a Potential Biocontrol Tool for Elimination of Malaria in Sub-Saharan Africa: Recommendations of a Scientific Working Group†. The American Journal of Tropical Medicine and Hygiene. 2018;98:1–49.

4. Thizy D, Emerson C, Gibbs J, Hartley S, Kapiriri L, Lavery J, et al. Guidance on stakeholder engagement practices to inform the development of area-wide vector control methods. PLoS Negl Trop Dis. 2019;13.

5. World Health Organization. WHO Guidance, Ethics and vector borne diseases [Internet]. 2020. Available from: https://apps.who.int/iris/bitstream/handle/10665/336075/9789240012738-eng.pdf

6. World Health Organization. Guidance framework for testing genetically modified mosquitoes, Second edition. 2021.

7. Hartley S, Thizy D, Ledingham K, Coulibaly M, Diabaté A, Dicko B, et al. Knowledge engagement in gene drive research for malaria control. PLoS neglected tropical diseases. 2019;13.

8. Kokotovich AE, Delborne JA, Elsensohn J, Burrack H. Emerging Technologies for Invasive Insects: The Role of Engagement. Annals of the Entomological Society of America. 2020;

9. Boëte C. Public engagement and communication: who is in charge? EMBO reports. 2017;19:e201745379.

10. Council H, Bureau HCB, Committee S. CEES recommendation concerning the referral dated 12 October 2015 on the use of mosquitoes with a biotechnologically modified genome to control disease vectors. 2017;

11. Schairer CE, Taitingfong R, Akbari OS, Bloss CS. A typology of community and stakeholder engagement based on documented examples in the field of novel vector control. PLoS Neglected Tropical Diseases. 2019;13:1–21.

12. Kolopack PA, Parsons JA, Lavery J V. What Makes Community Engagement Effective?: Lessons from the Eliminate Dengue Program in Queensland Australia. PLoS Neglected Tropical Diseases. 2015;9:1–19.

13. George DR, Kuiken T, Delborne JA. Articulating “free, prior and informed consent” (FPIC) for engineered gene drives. Proceedings of the Royal Society B: Biological Sciences. 2019;286.

14. Emerson C, James S, Littler K, Randazzo F. Principles for gene drive research. Science. 2017;358:1135–6.

15. Kuzma J, Gould F, Brown Z, Collins J, Delborne J, Frow E, et al. A roadmap for gene drives: using institutional analysis and development to frame research needs and governance in a systems context. Journal of Responsible Innovation. Routledge; 2018;5:S13–39.

16. African Union. Gene drives for malaria control and elimination in Africa. 2018.

17. Academy A, Science OF. Gene Drive Discussion Paper. 2017;

18. Kolopack PA, Lavery J V. Informed consent in field trials of gene-drive mosquitoes. Gates Open Research. 2017;1:14.

19. Singh JA. Informed consent and community engagement in open field research: lessons for gene drive science. BMC Medical Ethics. BMC Medical Ethics; 2019;20:1–12.

20. Wedell N, Price TAR, Lindholm AK. Gene drive: progress and prospects. Proceedings of the Royal Society B: Biological Sciences. Royal Society; 2019;286:20192709.

21. Roberts A, Thizy D. Ethical principles informing Target Malaria’s engagement strategy: Timely co-development with relevant communities - IN REVIEW. Malaria Journal. 2021;

22. Quinlan MM, Birungi J, Coulibaly MB, Diabaté A, Facchinelli L, Mukabana WR, et al. Containment Studies of Transgenic Mosquitoes in Disease Endemic Countries: The Broad Concept of Facilities Readiness. Vector-Borne and Zoonotic Diseases. Mary Ann Liebert, Inc., publishers; 2018;18:14–20.

23. Hartley S, Thizy D, Ledingham K, Coulibaly M, Diabaté A, Dicko B, et al. Knowledge engagement in gene drive research for malaria control. PLOS Neglected Tropical Diseases. 2019;13:e0007233.

24. Barry N, Toé P, Pare Toe L, Lezaun J, Drabo M, Dabiré RK, et al. Motivations and expectations driving community participation in entomological research projects: Target Malaria as a case study in Bana, Western Burkina Faso. Malaria Journal [Internet]. 2020;19:199. Available from: https://doi.org/10.1186/s12936-020-03277-7

25. Wanyama Chemonges E, Pare Toe L, Dicko B, Coulibaly M. Co‐developing a common glossary with stakeholders for engagement on new genetic approaches for malaria control in a local African setting. Malaria JournalMalaria Journal. 2021;

26. Thizy D, Pare Toe L, Mbogo C, Matoke-Muhia D, Alibu VP, Barnhill-Dilling SK, et al. Proceedings of an expert workshop on community agreement for gene drive research in Africa - Co-organised by KEMRI, PAMCA and Target Malaria. Gates Open Research. 2021;5:19.

27. Barbosa S, Pare Toe L, Thizy D, Vaz M, Carter L. Engagement and social acceptance in genome editing for human benefit : Reflections on research and practice in a global context [ version 1 ; peer review : 1 approved ] Sebastián Barbosa. 2020;1–13.

28. Bartumeus F, Costa GBGB, Eritja R, Kelly AHAH, Finda M, Lezaun J, et al. Sustainable innovation in vector control requires strong partnerships with communities. PLOS Neglected Tropical Diseases. 2019;13:e0007204.

29. Hayes KR, Barry S, Beebe N, Dambacher JM, Barro P De, Ferson S, et al. Risk Assessment for Controlling Mosquito Vectors with Engineered Nucleases: Sterile Male Construct Final report. 2015;196.
